# OVO Positively Regulates Essential Maternal Pathways by Binding Near the Transcriptional Start Sites in the *Drosophila* Female Germline

**DOI:** 10.1101/2023.11.01.565184

**Published:** 2023-12-08

**Authors:** Leif Benner, Savannah Muron, Jillian G. Gomez, Brian Oliver

**Affiliations:** 1. Section of Developmental Genomics, Laboratory of Biochemistry and Genetics, National Institute of Diabetes and Digestive and Kidney Diseases, National Institutes of Health, Bethesda, MD, USA. 2; 2. Department of Biology, Johns Hopkins University, Baltimore, MD, USA

## Abstract

Differentiation of female germline stem cells into a mature oocyte includes the expression of a number of mRNAs and proteins that drive early embryonic development in *Drosophila*. We have little insight into what activates the expression of these maternal factors. One candidate is the zinc-finger protein OVO. OVO is required for female germline viability, and has been shown to positively regulate its own expression, as well as a downstream target, *ovarian tumor* (*otu*), by binding to the transcriptional start site (TSS). To find additional OVO targets in the female germline and further elucidate OVO’s role in oocyte development, we performed ChIP-seq to determine genome-wide OVO occupancy, as well as RNA-seq to where OVO is required. OVO preferentially binds in close proximity to target TSSs genome-wide, is associated with open chromatin, transcriptionally active histone marks, and OVO-dependent expression. Motif enrichment analysis on OVO ChIP peaks identified a 5’-TAACNGT-3’ OVO DNA binding motif near TSS, but without the precise motif spacing relative to TSS characteristic of RNA Polymerase II complex binding core promoter elements. Integrated genomics analysis showed that 525 genes that are bound and increase in expression downstream of OVO are known to be maternally loaded into eggs and early embryos. These include genes involved in anterior/posterior/germ plasm specification (*bcd, exu, swa, osk, nos, pgc, gcl*), egg activation (*png, plu, gnu, wisp, C(3)g, mtrm*), translational regulation (*cup, orb, bru1, me31B*), and vitelline membrane formation (*fs(1)N, fs(1)M3, clos*). This suggests that OVO is a master transcriptional regulator of oocyte development and is responsible for the expression of structural components of the egg as well as maternally provided RNAs that are required for early embryonic pattern formation.

## Introduction

*Drosophila* early embryonic development is directed by events that take place during oogenesis. Germline stems cells asymmetrically divide to renew the stem cell population and send one daughter cell towards oogenesis. In the germarium, oogenesis is preceded by four rounds of incomplete mitotic divisions resulting in a 16 cell egg chamber. One cell is specified as the oocyte which is arrested in prophase of meiosis I, while the rest of the 15 cells enter endoreplication cycles and become nurse cells (NCs). Once the 16 cell egg chamber buds from the germarium, the NCs begin to transcribe and translate a vast array of RNAs and proteins that serve diverse functional roles (Bastock and St Johnston 2008; Spradling et al. 2022). These roles include positioning maternal mRNAs and proteins in the correct spatial orientation to support anterior/posterior, and dorsal ventral axis specification, as well negative regulators of translation to ensure that the maternal mRNAs are not translated before fertilization (Lasko 2012). The oocyte also contains a number of proteins and mRNAs that are needed for egg activation, completion of meiosis and initiation of embryonic development after fertilization (Avilés-Pagán and Orr-Weaver 2018). In order to repeat the process of oogenesis from generation to generation, germ cells in the developing embryo need to be specified and maintained separately from the rest of the developing somatic cell population. This requires maternal localization of the germ plasm and early pole cell formation in the embryo (Mahowald 2001). While thematic elements of this complex orchestration have been well studied, coordinate regulation of the symphony has not.

In particular, maternal control of stepwise embryonic pattern formation during development at the RNA and protein level has been extensively studied. However, the transcriptional control of these pathways during oogenesis are less well understood. Few positive regulators of female specific germ cell transcription have been identified. Genes such as *grauzone* (*grau*) and *maternal gene required for meiosis* (*mamo*) have been shown to activate the transcription of *cortex* and *vasa*, respectively, in the female germline (Harms et al. 2000; S. Nakamura et al. 2019). Active repression of male specific transcription through the activity of *egg, wde*, and *Su(var)205* (Smolko, Shapiro-Kulnane, and Salz 2018), or global repression of non-ovarian transcriptional networks through the function of *sov* (Leif Benner et al. 2019), have shown the importance of heterochromatin formation in the female germline for cellular identity and oocyte development. In fact, important recent work has shown the importance of transcriptional repression, mediated through changes in histone modifications, as a key regulator of egg chamber differentiation. GSCs have been shown to exist in a sort of ‘ground state’ of histone modifications. Characterized with modest non-canonical repressive H3K9me3 and H3K27me3 histone marks at many genes, as well as transcriptionally active H3K27ac histone marks and open chromatin at others (Pang et al. 2023; DeLuca et al. 2020). As oocyte development continues, repressive histone marks associated with heterochromatin begin to increase in abundance resulting in fewer histone marks associated with transcription and open chromatin. This suggests that gene expression becomes more restricted throughout the differentiation process. However, it is unlikely that the female germline directs oocyte development solely through a repressive transcriptional model. Whether the female germline expresses paralogs of the RNA polymerase II complex, like the male germline (M. Hiller et al. 2004; M. A. Hiller et al. 2001; Lu et al. 2020), or if there are pioneering transcription factors involved in determining the open chromatin status for female germ cell-specific expression, or something else entirely, has yet to be determined.

Although few female specific germline transcription factors have been identified, the conserved zinc-finger transcription factor *ovo* has long been known to be required for female germ cell viability. Female germ cells that are *ovo*^−^ do not survive into adulthood (Oliver, Perrimon, and Mahowald 1987; Oliver, Pauli, and Mahowald 1990; Benner et al. 2023). Hypomorphic *ovo* alleles, specifically ones that disrupt the transcriptional activator OVO-B, show an arrested egg chamber phenotype, indicating that wild-type OVO-B activity is required for oocyte maturation (Salles et al. 2002; Benner et al. 2023). Germline OVO is expressed at all stages of oogenesis, where it is eventually maternally loaded into the egg. Maternal OVO becomes specifically localized to the developing germline and persists throughout embryogenesis until zygotic OVO is expressed (Hayashi et al. 2017; Benner et al. 2023). Thus OVO is eternally expressed in the female germline, suggesting it may be a key regulator of female specific germline transcription. However, only two downstream targets have previously been identified for OVO. OVO has been shown to positively regulate the expression of its own transcription, therefore executing an autoregulatory loop, as well as positively regulating the transcription of the gene *ovarian tumor* (*otu*)(Lü et al. 1998; Bielinska et al. 2005; Lü and Oliver 2001; Andrews et al. 2000). *otu* is also required in the female germline, where *otu*^−^ germ cells show viability and germline tumor phenotypes (Bishop and King 1984). The *ovo* phenotype is epistatic to that of *otu*, and ectopic *otu* expression cannot rescue female germ cell death due to loss of *ovo* (Hinson, Pettus, and Nagoshi 1999; Pauli, Oliver, and Mahowald 1993). Therefore, *ovo* must be responsible for activating the transcription of genes in addition to *otu* for female germ cell survival and differentiation.

We expanded our knowledge of OVO’s role in the female germline by determining genome-wide OVO occupancy and global transcriptional changes downstream of OVO. This allowed us to determine which genes OVO binds and which genes transcriptionally respond to OVO *in vivo*. We show that OVO is directly regulating essential maternal pathways such as axis specification, primordial germ cell formation, egg activation, and maternal mRNA translation regulation. Together, we show that OVO plays a pivotal role in the positive transcriptional regulation of oocyte and early embryonic development. We show that OVO likely carries out this regulation by binding at or in close proximity to the promoters of the genes it regulates and that OVO DNA binding motifs are enriched at or near the transcriptional start site (TSS) of OVO responsive genes, although the spacing of OVO binding sites suggests that it is not a component of the RNA Polymerase complex. OVO binding is also a signature of open chromatin status and active transcription throughout oocyte differentiation. Altogether, we suggest that OVO is required for the activity of a large number of female germline promoters and is likely a key regulator of oocyte maturation and RNAs and proteins that are required for early embryonic development.

## Methods

All reagents used in this study can be found in the FlyBase recommended supplementary ART table (Table S1).

### Fly Husbandry, Transgenesis, and CRISPR/Cas9

All fly crosses were conducted at 25°C with 65% relative humidity and constant light unless otherwise noted. Flyfood consisted of premade flyfood (D20302Y) from Archon Scientific (Durham, NC).

### Immunofluorescence and Image Analysis

Adult females were collected and fed for 3-5 days before ovaries were dissected and fixed in 5.14% formaldehyde (Pierce, ThermoFisher Scientific) in phosphate buffered saline (PBS, Gibco, ThermoFisher Scientific) containing 0.1% Triton X-100 (Millipore Sigma)(PBTx) for 15 minutes. Ovaries were then washed 3 times for 5 min with PBTx followed by a blocking step in PBTx supplemented with 2% normal goat serum (NGS, Invitrogen, ThermoFisher Scientific) and 0.5% bovine serum albumin (BSA, Millipore Sigma)(BBTx) for 30 minutes. Primary antibodies were diluted to their appropriate concentrations in BBTx and ovaries were incubated in primary overnight at 4°C (rabbit anti-Vasa, 1:10K; mouse anti-α-Spectrin, 1:200; rat anti-HA, 1:100; mouse anti-BAM, 1:25; chicken anti-GFP, 1:500; mouse anti-ARM, 1:200; rabbit anti-MSL-2, 1:5K). The next day, ovaries were washed 3 times for 5 min with PBTx and incubated with appropriate secondary antibodies diluted (1:500) in BBTx at room temperature for 2 hours (Alexa Fluor goat anti-rat 488, Alexa Fluor goat anti-chicken 488, Alexa Fluor goat anti-rabbit 488, Alexa Fluor goat anti-mouse 568, Alexa Fluor goat anti-rat 568, Alexa Fluor goat anti-rabbit 633; Invitrogen,ThermoFisher Scientific). Ovaries were washed 3 times for 5 min with PBTx before incubation at room temperature in 1 μg/mL DAPI (Invitrogen, ThermoFisher Scientific) solution in PBS for 30 minutes. DAPI was then removed and ovaries were stored in PBS at 4°C until mounting. To mount, ovaries were transferred to a microscope slide before adding Ultramount Aqueous Permanent Mounting Medium (Dako, Agilent) and then coverslipped. Male testes were treated in the same manner, the only difference being that they were fixed in 4.5% paraformaldehyde in PBTx for 25 minutes. All steps were completed on a rotating nutator at room temperature unless otherwise noted.

Ovaries and embryos were imaged on a Zeiss 780 LSM confocal microscope (Carl Zeiss AG) using the Zen Black software (Carl Zeiss AG) . Image analysis was conducted using Fiji(Schindelin et al. 2012).

### RNA-seq library preparation and sequencing

Twenty 3-5 day old *ovo^ΔBP^*/*ovo^ovo-GAL4^*; *UASp-GFP* and *ovo^ΔBP^*/*ovo^ovo-GAL4^*; *UASp-3xFHA-OVO-B* ovaries were dissected and germariums through pre-vitellogenic egg chambers were removed with microdissection scissors and placed in ice cold PBS making up one biological replicate. RNA was then extracted from four biological replicates with a Qiagen RNeasy Plus Kit (Qiagen) according to the manufacturer’s protocol, eluted in dH_2_O, and RNA concentrations were measured with Quant-iT RiboGreen RNA Assay Kit (ThermoFisher Scientific). 500 ng of total RNA was then used to make RNAseq libraries with an Illumina Stranded mRNA Prep Kit according to the manufacturer’s protocol (Illumina). IDT for Illumina RNA UD Indexes Set A were used. Library concentrations were measured with Quant-iT PicoGreen dsDNA Assay Kit (ThermoFisher Scientific), pooled, and then 50 nucleotide paired-end DNA sequencing was completed on an Illumina NovaSeq 6000 system using a S1 flow cell (Illumina). Raw RNAseq reads are available at the SRA under accession (Not uploaded yet).

### ChIP-seq library preparation and sequencing

Adult *ovo^Cterm-3xFHA^* and *ovo^Cterm-GFP^* females were collected and fed for 24 hours before ovaries were dissected. 50 dissected ovaries were placed in ice cold phosphate buffered saline (PBS, Gibco, ThermoFisher Scientific) and then incubated in 1 mL crosslinking solution containing 2% formaldehyde (Pierce, ThermoFisher Scientific) (50mM HEPES Buffer, 1mM EDTA, 0.5 mM EGTA, 100mM NaCl), and rotated at 37°C for 20 minutes. Ovaries were then incubated in 1 mL stop solution (125mM Glycine, 0.01% Triton X-100 (Millipore Sigma), diluted in PBS) and rotated for 5 minutes at room temperature. Ovaries were then washed twice with 1 mL ice cold wash buffer (0.01% Triton X-100 in PBS) for 5 minutes. The last wash was removed and ovaries were stored at −80°C until future processing. Once all samples were collected, 4x50 ovaries were then homogenized in 250 μL RIPA lysis buffer (Pierce, ThermoFisher Scientific) containing 1x protease inhibitor cocktail (cOmplete Mini Protease Inhibitor Cocktail, Roche, Millipore Sigma) and 1 mM PMSF (Roche, Millipore Sigma) and kept on ice for 10 minutes. 40mg of 212-300 μm acid-washed glass beads (Millipore Sigma) were then added to homogenized ovary lysate. Samples were then sonicated with a Bioruptor Pico sonication device (Diagenode) at 4C for 15 cycles of 30 seconds on and 30 seconds off. Sonicated lysate was then transferred to a new tube and centrifuged at 13,300 rpm for 10 minutes at 4°C. Three supernatants were then combined to form one biological replicate. 100 μL for each biological replicate was removed and stored at −80°C for input control. To pull down C-terminally tagged OVO, 100 μL of monoclonal anti-HA-agarose (Millipore Sigma) or 50 μL of ChromoTek GFP-Trap agarose (Proteintech) were washed three times with RIPA lysis buffer and spun down at 1,200 RPMs for one minute at 4°C. 550 μL of *ovo^Cterm-3xFHA^* supernatant was added to monoclonal anti-HA-agarose and 550 μL of *ovo^Cterm-GFP^* supernatant was added to ChromoTek GFP-Trap agarose. Samples were supplemented with 1x protease inhibitor cocktail and 1 mM PMSF and incubated on a rotator at 4°C overnight.

The next day, agarose was washed in a stepwise fashion with solutions from a Chromatin Immunoprecipitation Assay Kit (Millipore Sigma), beginning with 1 mL of a low salt wash buffer, high salt wash buffer, LiCl buffer, and ending with 2 washes in 0.1x TE buffer. 300 μL of freshly prepared ChIP elution/decrosslinking solution (1% SDS, 100mM NaHCO_3_, 250 mM NaCl, 10mM EDTA, 50 mM Tris-HCl, 200 μg/mL Proteinase K) was added to the pelleted agarose, or 200 μL of chip elution/decrosslinking solution was added to 100 μL input control, and incubated at 65°C overnight. DNA was extracted by adding 300 μL phenol:chloroform:iso-amyl alcohol (125:24:1) (Millipore Sigma). The samples were vortexed for 30 seconds then centrifuged at 13,300 RPMs for 5 minutes at 4°C. The aqueous layer was extracted and this process was repeated once more. 1 μL glycogen (20 mg/mL), 30 μL 1M sodium acetate, and 750 μL 100% EtOH was added to the extracted aqueous layer, vortexed, and incubated at −20°C for 30 minutes. Solution was spun at 13,300 RPMs for 20 min at 4°C. Supernatant was removed and the pellet was washed with 500 μL 70% EtOH and spun down at 13,300 RPMs for 20 minutes at 4°C. This step was repeated but with 100% EtOH. The resulting pellet was briefly speedvacced and resuspended in 50 μL dH_2_O.

To make ChIP-seq libraries, DNA concentration for immunoprecipitated and input control samples were measured with a Quant-iT PicoGreen dsDNA Assay Kit (ThermoFisher Scientific). 5 ng of DNA for each sample was then used with the NEBNext Ultra II DNA Library Prep Kit for Illumina (New England Biolabs) and completed according to the manufacturer’s protocol. ChIP-seq library concentrations were then measured with a Quant-iT PicoGreen dsDNA Assay Kit, pooled, and then 50 nucleotide paired-end DNA sequencing was performed on an Illumina NovaSeq 6000 system using the XP workflow (Illumina). Raw ChIP-seq reads are available at the SRA under accession (Not uploaded yet).

### RNA-seq, ChIP-seq, CAGE-seq and Gene Ontology Analysis

For RNA-seq analysis of *ovo^ΔBP^*/*ovo^ovo-GAL4^*; *UASp-GFP* and *ovo^ΔBP^*/*ovo^ovo-GAL4^*; *UASp-3xFHA-OVO-B* ovaries, 50 nucleotide paired-end reads were mapped to the FlyBase r6.46 genome (Gramates et al. 2022) for differential expression analysis and the BDGP Release 6 Drosophila Genome (dos Santos et al. 2015) for read level genome browser tracks using Hisat2 (-k 1 --rna-strandness RF --dta)(Kim et al. 2019). DNA sequences for *GAL4* and *GFP* were added to the FlyBase r6.46 genome as separate chromosomes. Mapped reads were then sorted and indexed with Samtools (samtools sort and samtools index)(Danecek et al. 2021). Gene level readcounts were then derived with htseq-count (-s reverse -r pos)(Anders, Pyl, and Huber 2015) and used for differential expression analysis with DeSeq2 (Love, Huber, and Anders 2014). Genes with 0 mapped reads were removed from the DESeq2 analysis.

For ChIP-seq analysis of OVO-HA, OVO-GFP, OVO-HA input, and OVO-GFP input samples, 50 nucleotide paired-end reads were mapped to the FlyBase r6.46 genome for peak calling analysis and the BDGP Release 6 Drosophila Genome for read level genome browser tracks using Hisat2 (-k 1 --no-spliced-alignment -X 900). Mapped reads were sorted using Samtools (samtools sort and samtools index) and duplicate reads were removed with Picard (REMOVE_DUPLICATES=true)(broadinstitute, n.d.). Significant ChIP peaks were called for OVO-HA and OVO-GFP versus their respective input controls separately using Macs3 callpeak software (-g 1.2e8 -q 0.0001)(Zhang et al. 2008). Overlapping ChIP peaks for OVO-HA and OVO-GFP were then determined with bedtools intersect software (Quinlan and Hall 2010). Peak calling for GSC ATAC-seq (SRR24203655), 32c ATAC-seq (SRR24203650), GSC H3K27ac (SRR11084657), H3K4me3 (SRR11084658), H3K27me3 (SRR11084656), H3K9me3 (SRR24203631), 8c NC H3K4me3 (SRR24203629), 32c NC H3K27ac (SRR24203635), and H3K27me3 (SRR11084652) ChIP-seq versus their respective input controls (SRR11084655, SRR11084651, SRR24203634, SRR24203637) was conducted in the same manner as OVO ChIP-seq.

In order to generate gene level read coverage tracks, deepTools’ bamCompare software was used to generate a single bigWig file comparing all replicates versus input controls (-bs 5 --effectiveGenomeSize 142573017 --normalizeUsing BPM --exactScaling --scaleFactorsMethod None)(Ramírez et al. 2016). The bigWig file was then uploaded to UCSC genome browser for visualization (Kent et al. 2002).

To generate read coverage plots centered on the motif location or OVO peak maximums, genomic locations of significant scoring motifs or peak maximums within overlapping OVO ChIP peaks were determined and used as input for deepTools’ computeMatrix reference-point (-a 2000 -b 2000 -bs 25 -- missingDataAsZero). Read density profiles for each motif or OVO peak maximum were then visualized with deeptools plotProfile. In order to generate read coverage plots centered on the TSS, the same methods as above were conducted except genes overlapping OVO ChIP peaks containing the respective significant OVO DNA binding motifs were used as input instead.

In order to generate gene level read coverage heatmaps, deepTools’ computeMatrix scale-regions software was used to generate a single matrix for genes that were bound by OVO (-bs 25 -- missingDataAsZero -m 4000 --metagene). This matrix was then used as input for deepTools’ plotHeatmap software to generate heatmaps of ChIP-seq and RNA-seq for the given OVO binding profiles centered on the TSS (--sortUsing max).

For CAGE-seq analysis, CAGE-seq libraries for ovary (SRR488283, SRR488282), testes (SRR488284, SRR488308, SRR488285, SRR488309), and male and female digestive system (SRR488289, SRR488288) tissues were downloaded and combined for each tissue type from the SRA. Reads were mapped to the BDGP Release 6 Drosophila Genome with Hisat2 (-k 1). Mapped reads were sorted with Samtools. Significant dominant TSSs were then determined with CAGEr software(Haberle et al. 2015) from sorted BAM files with getCTSS and annotated with annotateCTSS using the dm6.ensGene.gtf file(Hubbard et al. 2002) downloaded from UCSC(Kent et al. 2002). CAGE-seq reads were normalized with normalizeTagCount (ce, method = “simpleTpm”, fitInRange = c(5, 40000), alpha = 1.15, T = 1*10^6) and then TSS clusters were determined with clusterCTSS (ce, threshold = 1, thresholdIsTpm = TRUE, nrPassThreshold = 1, method = “paraclu”, maxDist = 20, removeSingletons = TRUE, keepSingletonsAbove = 5) in order to determine the dominant significant TSS for each respective tissue.

Gene ontology enrichment analysis was completed with g:Profiler’s g:GOSt software (Raudvere et al. 2019) on the set of genes overlapping OVO ChIP peaks over the TSS, significantly upregulated in the presence of OVO, and contained significant OVO DNA binding motifs. Default parameters were used for the enrichment analysis and only GO biological process terms were searched for enrichment with the gene list.

Fisher’s exact test was conducted for each respective analysis with the fisher.test() command in R (R Core Team 2021).

### *de novo* Motif Enrichment and Promoter Motif Analysis

DNA sequences from significant overlapping OVO ChIP peaks were extracted from the Drosophila r6.46 genome and submitted to STREME software (Bailey 2021) of the MEME suite (Bailey et al. 2015). The default parameters were used for *de novo* motif enrichment analysis. After identifying ‘OVO Motif One’, OVO ChIP peaks that contained that sequence were removed and the resulting ChIP peaks were resubmitted for STREME analysis deriving derivative OVO DNA binding motifs. Significant OVO DNA binding motifs and *in vitro* OVO DNA binding motifs were searched in the BDGP Release 6 Drosophila Genome using FIMO (Grant, Bailey, and Noble 2011). In order to find significant DNA binding motif matches for ‘OVO Motif One’, this motif from STREME was submitted to Tomtom software (Gupta et al. 2007) of the MEME suite and searched within the JASPAR Core Insect database (2022)(Castro-Mondragon et al. 2022).

Promoter motif analysis was conducted by extracting the DNA sequences 200 nucleotides upstream and downstream of the significant dominant TSSs from CAGE-seq analysis for each respective tissue type. All common core promoter motifs (FitzGerald et al. 2006; Ohler et al. 2002) were then searched in these sequences depending on their strand specificity with the use of FIMO from the MEME suite using a p-value of < 0.003 for all non-OVO promoter motifs. All OVO motifs found in this study and through *in vitro* methods were also searched with the same method, except a p-value of < 0.0002 was used.

## Results

### OVO Binds Promoters Genome Wide

OVO-B, the predominant protein isoform expressed from the *ovo* locus in the female germline (Benner et al. 2023), is a positive regulator of transcription (Andrews et al. 2000). OVO-B positively regulates the gene expression of *otu* and *ovo* transgenic reporter constructs require OVO binding sites both at and upstream of the TSS in order to recapitulate full reporter expression (Bielinska et al. 2005; Lü et al. 1998; Lü and Oliver 2001). Females hemizygous for antimorphic dominant gain-of-function (*ovo^D^*) or homozygous recessive (*ovo^D1rv^*) *ovo* alleles lack germ cells in the adult ovary (Oliver, Perrimon, and Mahowald 1987; Benner et al. 2023). True OVO-B null alleles created by deletion of the *ovo-B* promoter have the same germ cell loss phenotype (Benner et al. 2023). The phenotypes of *otu* females range from germ cell death to ovarian tumors depending on the allele and undefined stochastic factors (Bishop and King 1984). It is possible that the germ cell death phenotype in *ovo*^−^ female germ cells can solely be explained by failure of OVO to activate *otu* expression, however, this is highly unlikely. The *ovo^D1rv^* phenotype is epistatic to *otu*^−^, and ectopic *otu*^+^ expression in *ovo*^−^ germ cells does not rescue the germ cell death phenotype (Hinson, Pettus, and Nagoshi 1999). This is not terribly surprising, as we know of no examples of a transcription factor that regulates a single gene. This suggests that OVO regulates the expression of additional genes in the female germline.

**Figure 1:**
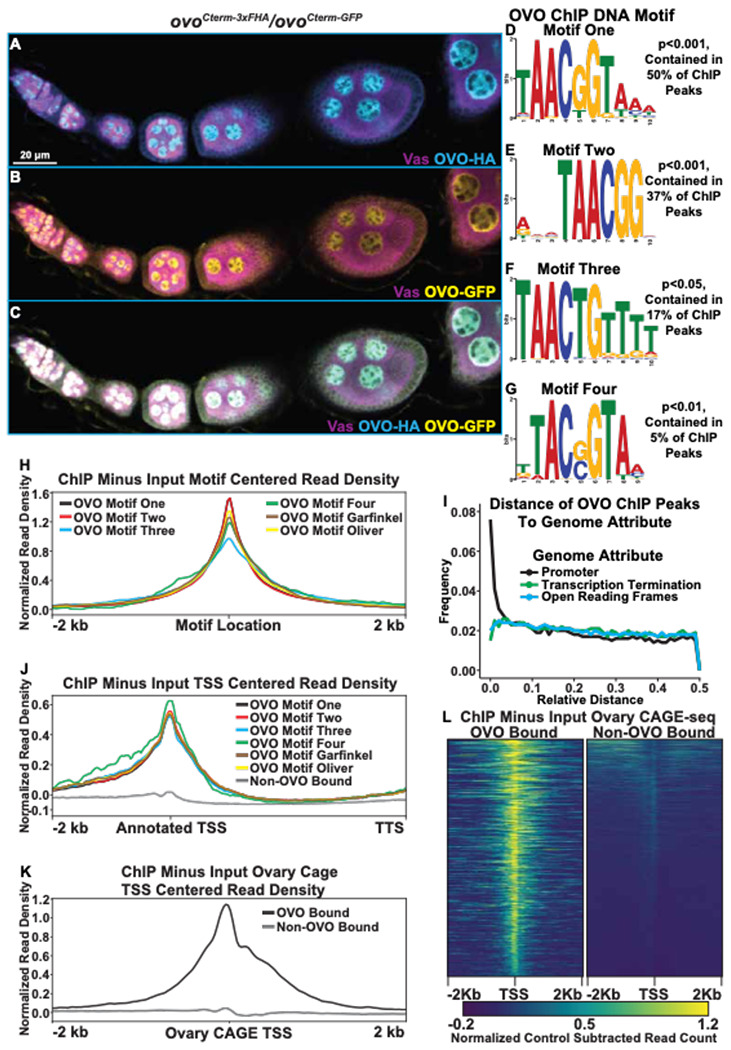
Significantly Enriched OVO DNA Binding Motifs and OVO ChIP Attributes Genome-Wide. A-C) Immunofluorescent staining of adult ovarioles of *ovo*^*Cterm-3xFHA*^*/ovo*^*Cterm-GFP*^ females (20x, scale bar = 20 μm). Ovarioles were stained for Vas (purple) to label the germline, HA (cyan) to label OVO-HA, and GFP (yellow) to label OVO-GFP. The homozygous version of these alleles were used to ChIP OVO. D-G) Significantly enriched motifs found within overlapping OVO ChIP peaks. The percentage of OVO ChIP peaks each motif was found and their corresponding p-value are indicated to the right. H) OVO ChIP minus input control ChIP-seq read coverage density centered on each individual motif’s location. I) Relative distance of OVO ChIP peaks to gene level promoters, terminations sequences, and open reading frames genome-wide. J) OVO ChIP minus input control ChIP-seq read coverage density for genes containing significant OVO ChIP peaks and the corresponding OVO DNA binding motif. Genes are centered on the transcriptional start site. K, L) OVO ChIP minus input control ChIP-seq read coverage density and heatmap plots centered on the dominant significant ovary CAGE-seq TSSs overlapping or not overlapping OVO ChIP peaks.

We wanted to identify the full stable of OVO target genes in the female germline by using two complementary genome-wide approaches to test for OVO presence and function. Specifically, we determined OVO occupancy genome-wide with ChIP-seq, and determined *ovo* function by comparing the RNA expression profiles between *ovo*^+^ and *ovo* hypomorphs in the female germline. In order to determine OVO occupancy genome-wide, we performed ChIP-seq on newly eclosed adult ovaries in triplicate, using two C-terminally tagged alleles as affinity purification tools (*ovo^Cterm-3xFHA^* and *ovo^Cterm-GFP^*, Figure 1A–C)(Benner et al. 2023). We compared immuno-purified OVO associated DNA with input DNA as a control, for a total of 12 ChIP-seq libraries, which we sequenced using the Illumina system. After quality control and alignment to the *Drosophila* r6.46 genome (Gramates et al. 2022), we used MACS3 (Zhang et al. 2008) to call significantly enriched peaks from OVO-HA and OVO-GFP compared to their respective input controls (see methods).

We first compared the pulldown results with the OVO-HA versus OVO-GFP ChIP reagents. The GFP pull down appeared to be more efficient, but never-the-less we found excellent agreement, as most OVO-HA peaks were also found in the OVO-GFP dataset. The OVO-GFP ChIP dataset had 7,235 significant ChIP peaks according to peak enrichment analysis derived from MACS3 genome-wide, while OVO-HA ChIP dataset had 3,393 significant peaks genome-wide (Table S2). To determine the similarity in significant peak calling between the two datasets, we calculated a Jaccard index (intersection/union) between the significantly enriched peaks from the tagged *ovo* allele bearing ovaries. The Jaccard index between OVO-HA and OVO-GFP ChIP peaks was 0.64 (where 0 = no overlap and 1 = full overlap) with a total of 3,094 ChIP peaks overlapping. Thus, almost all significant OVO-HA ChIP peaks were also found within the OVO-GFP ChIP dataset (91% of OVO-HA peaks overlapped OVO-GFP peaks). OVO-GFP pulldown was either more effective, or less likely, promiscuous. Regardless, we decided to use the conservative intersection of the two datasets (3,094 peaks) for downstream OVO occupancy informatics (Table S3).

OVO is a sequence-specific DNA-binding protein, but many transcription factors also have a more general affinity for DNA, additionally, immunoprecipitation can capture indirect interactions due to nuclear topology in addition to direct sequence-specific binding. For example, in the particular case of OVO TSS binding, this could be direct, as shown in the case of *ovo-B* and *otu* loci (Lü et al. 1998; Lü and Oliver 2001; Bielinska et al. 2005), or could be due to looping of an OVO-bound enhancer to the core promoter. Determining if there are canonical OVO binding sites at peaks can help to distinguish direct and indirect binding. If OVO is directly binding to the TSS, we would expect to find OVO binding site enrichment at that location. To examine the sequences enriched in peaks, we looked directly for the known OVO binding sites previously defined by footprinting (Lü et al. 1998) and SELEX-seq ((Bielinska et al. 2005; Lee and Garfinkel 2000). We also did *de novo* motif finding on this substantial dataset to refine the sequence specific motifs bound by OVO and perhaps other motifs associated with other transcription factors preferentially bound by OVO enhancers, or OVO proximal sites in 3D nuclear space. We performed novel motif enrichment analysis using STREME (Bailey 2021) with our overlapping ChIP peaks and found a number of significant motifs within our dataset. The most significant motif, 5’-TAACGGTAAA-3’ was found in 50% of significant ChIP peaks (Figure 1D, ‘Motif One’). This motif is highly similar to the OVO DNA binding motif that has been reported twice before, 5’-AGTAACNGT-3’ (SELEX method, ‘Garfinkel OVO Motif’) and 5’-TGTAACNGT-3’ (Footprinting method, ‘Oliver OVO Motif’). The only differences between motif one in our dataset and the literature, is that the *de novo* motif is two nucleotides shorter than the previously described motifs at the 5’ end, extends 3 nucleotides downstream, and includes a second G near the 3’-end (Figure S1A). Collectively, this is strong evidence that the core OVO binding sequence is 5’-TAACGGT-3’. Some binding sites can be recognized by multiple transcription factors. To determine if other characterized transcription factors recognize this sequence, we searched for significant matches to motif one in the Jaspar database (Castro-Mondragon et al. 2022) of known *Drosophila* motifs using Tomtom (Gupta et al. 2007). The OVO DNA binding motif (‘Garfinkel OVO Motif’) was scored as a significant match (Figure S1A, p<0.05). While there may well be other TFs binding near OVO, we did not identify them based on known binding site data.

Not every peak region had one of these consensus OVO motifs. This does not mean *a priori* that they bound OVO indirectly. Motif enrichment can be driven by a few strongly enriched sequences, so that more minor enrichments of variants are missed. Therefore, we carefully examined the 50% of our overlapping OVO ChIP peaks where motif one was not found. This second round of *de novo* analysis revealed enrichment of OVO DNA binding motif derivatives. For example, the third most significant motif (found in 37% of peaks) was 5’-RWMTAACGGV-3’ (Figure 1E, motif two). This motif had the core 5’-TAACGGT-3’ sequence found in all three aforementioned methods, however, the last nucleotide in the core sequence is unspecific and lacks the three 3’ nucleotides found in motif one. Two other derivative motifs, 5’-TAACTGTTTT-3’ (found in 17% of sequences, Figure 1F, motif three), and 5’-TTACSGTAA-3’ (found in 5% of sequences, Figure 1G, motif four), vary within the central core motif (at positions 5 and 2 of the core sequence, respectively) and upstream and downstream ends. Searching for all variations of the OVO DNA binding motif (Table S4) within our significant overlapping ChIP peaks indicated that 72% of peaks contained at least one variation of these four binding motifs. It is a reasonable hypothesis that OVO peaks are most often due to direct, rather than indirect OVO binding.

A prediction for direct OVO binding to motifs, is that the motif should be centered within the peak of fragmented input DNA sequences. Therefore, we plotted the significant ChIP (minus input) read density peaks centered on the location of the motif itself. We found that the read density for all ChIP peaks that contain each one of the *de novo* OVO motifs, as well as the *in vitro* OVO motifs, are centered over the motif location (Figure 1H). This suggests that all of these motifs from our analysis are bonafide OVO DNA binding sites *in vivo*. While it is possible that OVO comes into contact with regions of DNA in three dimensional nuclear space non-specifically, the presence of OVO motifs within a large percentage of significant ChIP peaks *in vivo*, and the ChIP peak read densities centered over the location of the motifs, strongly reinforces the idea that our dataset contains regions centered on sequence-specifically bound OVO transcription factor in the ovary.

Given the clear function of OVO occupancy near the TSS of its two known targets, *ovo* and *otu* (Lü et al. 1998; Bielinska et al. 2005; Lü and Oliver 2001), we were interested in determining if OVO peaks are generally near the TSS of other target genes as well. In addition to informing the biology of OVO function, this simplifies the problem of associating peaks to potential functional target genes. As a preliminary test of this idea, we determined if the fully overlapping OVO-HA and OVO-GFP peaks were spatially enriched with respect to the currently annotated gene model elements such as TSS, open reading frames (ORFs), or transcription termination sequences (TTS). If the TSS association of OVO at the two known targets reflects a general propensity, then we expected OVO ChIP peaks to be more closely associated with TSS than other gene elements. To carry out this analysis, we normalized the genome for these three gene elements, such that the distance between adjacent loci was 1. If there is no enrichment for OVO peaks to a specific gene element, then the peak location would have an equal frequency from 0.0 to 0.5 relative distance. Measuring the relative distance of our OVO ChIP peaks to TSS, ORFs, and TTS, showed that the OVO binding was highly enriched near TSS/promoter locations and was not correlated with ORF and TTS locations (Figure 1I). These results confirmed that OVO is characterized by core promoter proximal binding. Since OVO ChIP peaks as a class are associated with TSS, we plotted the ChIP minus input read density of genes that overlap significant ChIP peaks to examine the full distribution. We found that the OVO ChIP read density was highly enriched over the TSS and was not due to a few highly enriched examples (Figure 1J). This builds on the idea that OVO is binding directly over, or in close proximity to the TSS of it’s target genes genome-wide. In other words, the previous work showing OVO binding the *ovo-B* and *otu* TSS (Bielinska et al. 2005; Lü et al. 1998; Lü and Oliver 2001) is typical. This very specific binding of OVO to the TSS is intriguing and unusual, as this region associates with the basal transcriptional machinery. It raises the possibility that OVO is not a typical transcription factor that acts primarily via enhancer binding, but might be part of the core promoter binding complex or acts to precondition the core promoter region for example.

The OVO ChIP read density was highly enriched over the annotated TSS of target genes, but TSS annotation is challenging and can be tissue specific. We were interested in empirically determining if the same enrichment was present in TSSs utilized specifically in ovarian tissue. In other words, where exactly does transcription start at these genes. The 5’ ends of mRNA are capped. In order to determine where these caps mapped to the genome, we analyzed used Cap Analysis of Gene Expression (CAGE-seq) data from adult *Drosophila* ovaries (SRR488283, SRR488282)(Chen et al. 2014) with CAGEr software (Haberle et al. 2015) and extracted the dominant significant TSSs in the ovary. CAGEr predicted 6,856 significant TSSs in the ovary dataset, of which 1,047 overlapped with OVO ChIP peaks. We plotted the OVO ChIP minus input read density centered on the significant ovary CAGE-seq TSSs for TSSs that overlapped or did not overlap OVO ChIP peaks (Figure 1K, 1L). We found that OVO ChIP read density was highly enriched over the location of TSSs from ovary CAGE-seq that overlapped OVO ChIP peaks when compared to TSSs that did not overlap OVO ChIP peaks. Thus, OVO TSS binding is not due to poor annotation of ovarian TTSs. Furthermore, OVO is binding at or near TSS of genes actively being transcribed in the ovary.

### OVO Binding is Associated with Open Chromatin and Transcriptionally Active Histone Marks

Our OVO ChIP data indicated that OVO was binding at or in close proximity to promoters genome-wide. OVO could have positive and/or negative effects on transcription at that location. For example, OVO could help recruit or sterically hinder RNA Polymerase binding to TSSs. Previous OVO reporter constructs show positive effects of OVO binding near TSS (Lü and Oliver 2001; Lü et al. 1998; Bielinska et al. 2005). If OVO binding is generally promoting transcription, then we hypothesize that it would be more closely associated with histone marks associated with active transcription, such as H3K27ac and H3K4me3, as well as lower nucleosome density that can be measured through ATAC-seq. In contrast, OVO binding would be expected to negatively correlate with repressive H3K9me3 and H3K27me3 histone marks and higher nucleosome density. It is technically difficult to determine changes in chromatin status and transcription in germ cells that lack OVO, as the phenotype is cell death (although we will return to this later for transcription profiling), but analyzing OVO binding in the context of ovarian chromatin was highly informative.

Recent work profiling nucleosome density and histone marks have shown that female GSCs have a ‘ground state’ chromatin profile (DeLuca et al. 2020), similar to the histone mark profiles that are found in early embryos (X.-Y. Li et al. 2014). This has been characterized to contain non-canonical H3K27me3 profiles and low H3K9me3 histone levels (Pang et al. 2023; DeLuca et al. 2020). As egg follicles differentiate, nurse cells begin to accumulate H3K9me3 marks, and H3K27me3 histone marks begin to accumulate over more traditional polycomb domains. This in turn leads to a decrease in the number of open chromatin peaks as well as H3K27ac histone marks, which are generally associated with active transcription (Pang et al. 2023; DeLuca et al. 2020). Essentially, these data support the idea that egg chambers restrict gene expression competency as they differentiate.

**Figure 2:**
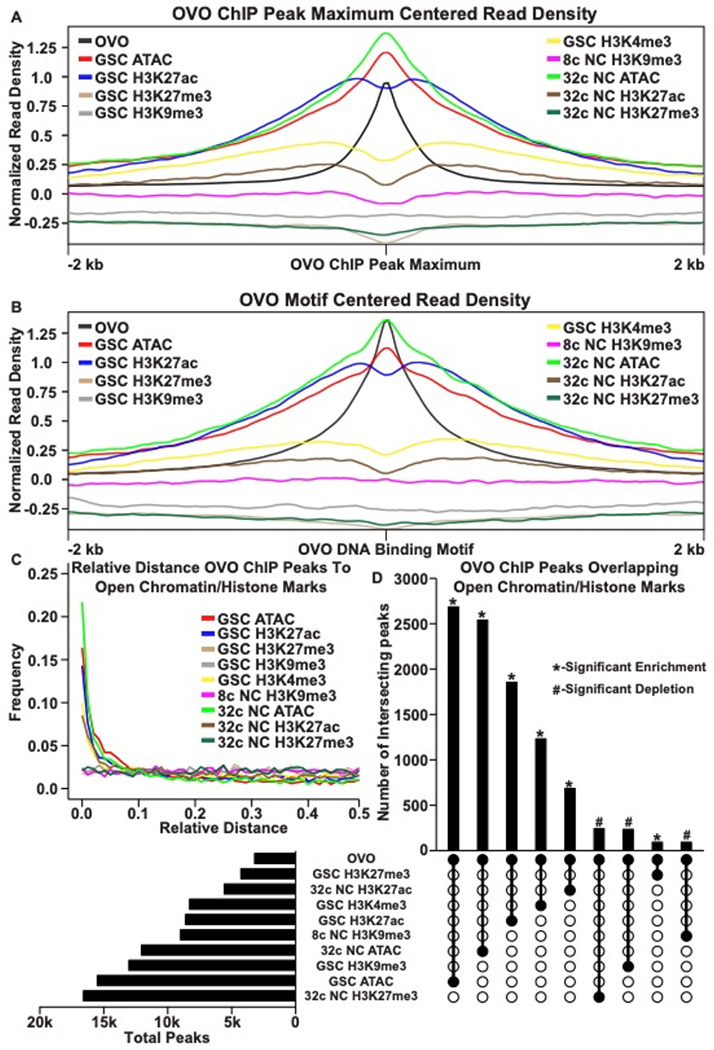
OVO DNA Binding is Associated with Open Chromatin and Transcriptionally Active Histone Marks. A, B) OVO ChIP minus input control, GSC and 32c ATAC-seq, GSC H3K27ac, H3K4me3, H3K27me3, H3K9me3, 8c NC H3K9me3, 32c NC H3K27ac, and H3K27me3 ChIP-seq read coverage density centered on each OVO peak maximum or individual motif’s location. C) Relative distance of OVO ChIP peaks to significantly called peaks for GSC and 32c ATAC-seq, GSC H3K27ac, H3K4me3, H3K27me3, H3K9me3, 8c NC H3K9me3, 32c NC H3K27ac, and H3K27me3 ChIP-seq genome-wide. D) Total number of significant peaks and the total number of overlapping peaks between OVO ChIP and GSC and 32c ATAC-seq, GSC H3K27ac, H3K4me3, H3K27me3, H3K9me3, 8c NC H3K9me3, 32c NC H3K27ac, and H3K27me3 ChIP-seq. Asterisk indicates significantly enriched overlap while hashtag indicates significantly depleted overlap between datasets.

In order to determine the relationship in our OVO ChIP data and other chromatin marks, we analyzed GSC H3K27ac, H3K27me3, H3K9me3, H3K4me3, and ATAC-seq data (Pang et al. 2023; DeLuca et al. 2020) with the same parameters used to establish significant OVO peaks in our OVO ChIP dataset. Our OVO ChIP data was from one day old ovaries and we did not profile specific follicle stages. So we also analyzed 32c (roughly stage 5 egg chambers) H3K27ac, H3K27me3, ATAC-seq, and 8c H3K9me3 (32c was not available) histone marks (Pang et al. 2023; DeLuca et al. 2020) to see if there were any stage specific differences in comparison to OVO DNA binding. We first plotted the read density of each respective chromatin mark minus their input control centered on either the OVO ChIP peak local maximum (Figure 2A) or OVO DNA binding motifs (Figure 2B). GSC ATAC and H3K27ac read density showed a high degree of enrichment over OVO ChIP peak maximums (Figure 2A) and OVO DNA binding motifs (Figure 2B), consistent with positive transcriptional activity. GSC H3K4me3 read density was, to a lesser extent, also enriched with OVO ChIP peak maximums and OVO DNA binding motifs. However, there was no read density enrichment for repressive GSC H3K27me3 and H3K9me3 histone marks. Since there was a high degree of read density enrichment between OVO ChIP and other chromatin marks/low nucleosome density we wanted to determine the extent of the overlap between significant OVO ChIP peaks and significantly called peaks from the different types and stages of histone marks and ATAC-seq data. In order to do this, we measured the relative distance of OVO ChIP peaks to the same datasets described above. We found that OVO ChIP peaks had a lower relative distance, and thus were spatially overlapping/closer in the genome, to 32c NC ATAC, GSC ATAC, GSC H3K27ac, GSC H3K4me3, and 32c NC H3K27ac peaks, in that order (Figure 2C). While the relative distance between OVO ChIP peaks and H3K9me3 and H3K27me3, regardless of stage, showed no spatial association. There was also further support for this association with transcriptionally active histone marks/open chromatin when measuring the overlap between significant OVO ChIP peaks and the respective significant histone ChIP and ATAC-seq peaks (Figure 2D). A Fisher’s exact test indicated a significant enrichment in overlapping peaks genome-wide between OVO and GSC ATAC (p < 0.001, odds ratio = 75.9), 32c NC ATAC (p < 0.001, odds ratio = Infinite, i.e. all OVO peaks overlapped ATAC-seq peaks), GSC H3K27ac (p < 0.001, odds ratio = 31.7), GSC H3K4me3 (p < 0.001, odds ratio =12.0), and 32c NC H3K27ac (p < 0.001, odds ratio = 7.9) peaks. While there was a significant depletion in overlapping peaks genome-wide between OVO and 32c NC H3K27me3 (p < 0.001, odds ratio = 0.6), GSC H3K9me3 (p < 0.001, odds ratio = 0.7), and 8c NC H3K9me3 (p < 0.001, odds ratio = 0.5). Altogether, suggesting that OVO binding genome-wide is tightly associated with open chromatin regardless of germ cell stage, and active transcription in GSCs. In other words, chromatin state data suggests OVO is acting positively on it’s target genes and raises the possibility that OVO-binding and open chromatin precede transcription.

### OVO DNA Binding Motifs are Evenly Distributed Around Promoters and are Enriched for INR, DPE, and MTE Elements

Our data thus far clearly indicates that OVO binding occurs at or very near the core promoter, a region recognized by an enormous collection of factors that associate with RNA polymerase to initiate transcription (Aoyagi and Wassarman 2000; Vo Ngoc, Kassavetis, and Kadonaga 2019). If these promoter proximal OVO sites are functional, they could be part of that complex, or could help facilitate the binding of that complex. The highly organized polymerase complex has sequence-specific DNA recognition sites with incredibly precise spacing between them, with an overall DNA footprint of a little less than 100bp (Rice, Chamberlin, and Kane 1993; FitzGerald et al. 2006; Ohler et al. 2002). If OVO binding sites at core promoters share this precision in spacing with other core promoter elements, then it is likely to be part of the complex. If not, then OVO is more likely to facilitate binding of the basal transcriptional machinery. Because of the extended footprint of engaged RNA polymerase, OVO and the basal machinery would not be likely to occupy the same region at the same time.

**Figure 3:**
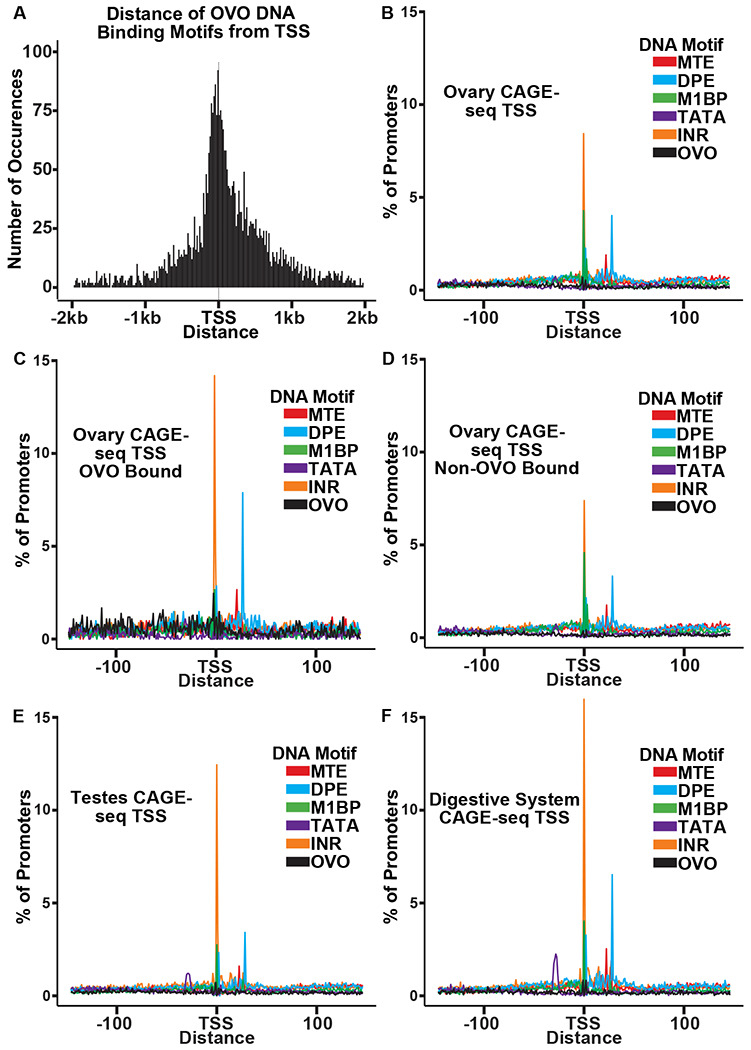
OVO Bound Promoters are Enriched for INR, DPE, and MTE elements. A) Histogram of the distance of *in vivo* and *in vitro* OVO DNA binding motifs within significant overlapping OVO ChIP peaks from the closest genes TSS. B-F) Histogram of the percent of promoters from tissue specific CAGE-seq analysis of common promoter motif elements centered on the dominant significant TSS.

Core promoters have sets of sequences with tightly constrained inter-motif distances that bind components of the transcription initiation complex. There are upstream binding sites such as TATA, sites at transcription start, such as the initiator (INR), and downstream promoter elements (DPE) (Vo Ngoc, Kassavetis, and Kadonaga 2019). The combinations of these elements is not random in mammals and *Drosophila (FitzGerald et al. 2006)*, and the families of motif combinations at the TSS of genes expressed in *Drosophila* are conserved over tens of millions of years of evolution (Chen et al. 2014). Like OVO chip peaks, OVO DNA binding motifs were highly enriched at or near the TSS (Figure 3A). We carefully analyzed the spacing of these sites relative to core promoter elements to see if spacing was precise at the nucleotide level.

We first searched for the presence of previously defined DNA motifs that are enriched at promoters (FitzGerald et al. 2006; Ohler et al. 2002; Lim et al. 2004) using FIMO (Grant, Bailey, and Noble 2011). We defined promoters by using the DNA sequences 150 nucleotides upstream and downstream of the significant dominant TSSs in our previously analyzed ovary CAGE-seq datasets (Chen et al. 2014). After extracting these sequences and searching for significant scoring motifs, we plotted the density of each motif in relation to the empirically mapped TSSs (Figure 3B). We also searched for all OVO motifs found in our significant ChIP peaks within these promoter sequences. When plotting the density of DNA motifs found in ovary CAGE-seq promoters, we found that there were prominent peaks for INR and M1BP (M1BP (J. Li and Gilmour 2013) = Ohler 1 (Ohler et al. 2002) = DMv4 (FitzGerald et al. 2006)) near the TSS, and MTE (Lim et al. 2004) and DPE elements downstream of the TSS. This distribution and frequency is consistent with the constrained location of these DNA motifs (FitzGerald et al. 2006; Ohler et al. 2002; Chen et al. 2014). Significantly, the OVO DNA binding motifs showed a broad distribution upstream and downstream of the TSS.

The precise core promoter architecture of OVO bound TSSs is revealed in the CAGE-seq dataset. Plotting the distribution of classical core promoter sequence elements in OVO bound promoters showed a similar, but exaggerated, profile compared to all care promoters of the ovary CAGE-seq dataset. We found a significant enrichment for INR (p < 0.01, odds ratio = 1.70), DPE (p < 0.01, odds ratio = 1.81), MTE (p < 0.01, odds ratio = 1.65), and most importantly, OVO DNA binding motifs (p < 0.01, odds ratio = 4.83), in ovary promoters that overlapped an OVO ChIP peak in comparison to the subset of ovary promoters that did not overlap an OVO ChIP peak (Figure 3C, 3D). This indicates that OVO bound promoters are more likely to contain these specific promoter elements than non-OVO bound promoters. As has been described before, promoters containing INR and DPE, but lacking TATA-box elements, are common among *Drosophila* gonad promoters compared to promoters of other tissue types (Chen et al. 2014). The presence of TATA-box elements is negatively associated with germline-specific gene expression (FitzGerald et al. 2006). We found that TATA-box elements were significantly depleted in ovary CAGE-seq promoters when compared to testes (p < 0.01, odds ratio = 0.78)(Figure 3E) or digestive system (p < 0.01, odds ratio = 0.50)(Figure 3F) CAGE-seq promoters. Indeed, both *ovo* and *otu* have TATA-less promoters. Briefly, OVO bound promoters are characterized by the presence of INR, DPE, MTE, and, of course, OVO DNA binding motifs. This could represent a functional class of promoters utilized for gene expression in the *Drosophila* ovary. Importantly, the distribution of OVO DNA binding motifs in ovary promoters is not fixed relative to TSSs or other core promoter elements. Thus, it is highly unlikely that OVO acts as a female germline RNA Polymerase complex member that anchors the complex to the core promoter and helps determine the +1 mRNA nucleotide. Rather, the imprecise location of OVO binding sites might suggest that OVO is more likely to facilitate the binding of other basal transcriptional factors.

### OVO Activates Gene Expression in the Female Germline

**Figure 4:**
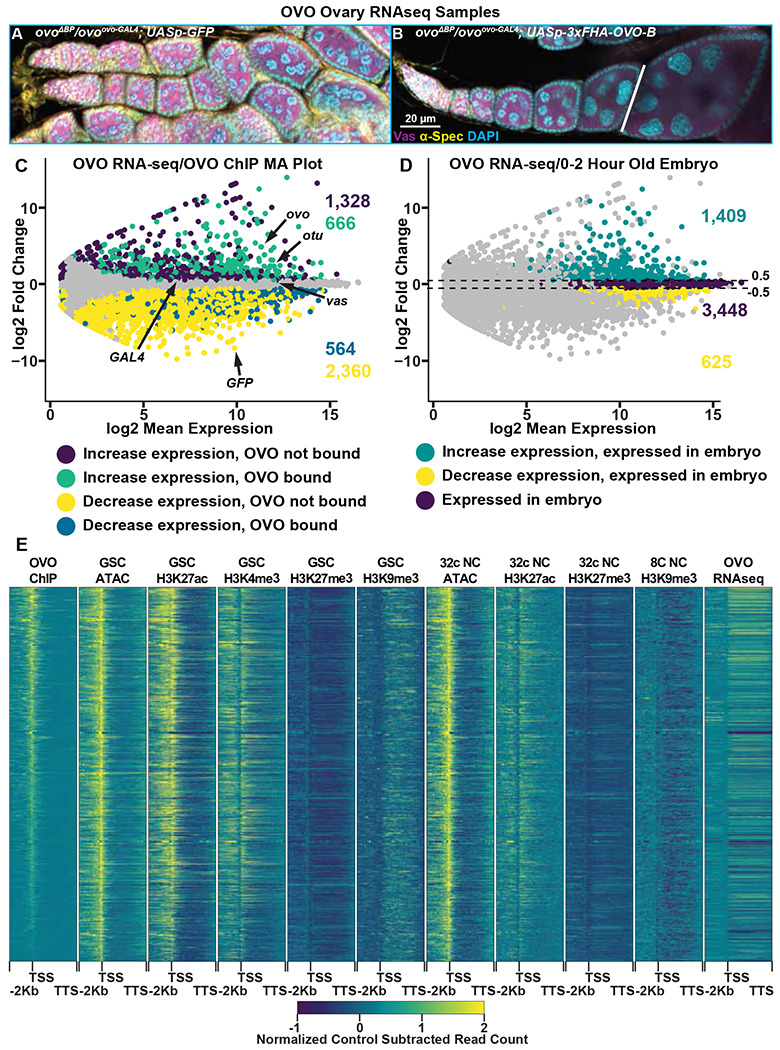
Genes Bound by OVO Increase in Expression in the Presence of OVO Genome-Wide. A-B) Immunofluorescent staining of adult ovarioles of the indicated genotypes (20x, scale bar = 20 μm). Ovarioles were stained for Vas (purple) to label the germline, α-Spectrin (yellow) to label dot spectrosome and fusomes, and DAPI (cyan) to label nuclei. Line indicates the dissection point for germarium through previtellogenic RNA-seq samples. C) MA plot of *ovo*^*ΔBP*^*/ovo^ovo-GAL4^*; *UASp-3xFHA-OVO-B* versus *ovo^ΔBP^/ovo^ovo-GAL4^*; *UASp-GFP* RNA-seq differential expression results. Purple dots indicate genes that significantly increased in expression and were not bound by OVO, cyan dots indicate genes that significantly increased in expression and were bound by OVO, yellow dots indicate genes that significantly decreased in gene expression and were not bound by OVO, blue dots indicate genes that significantly decreased in gene expression and were bound by OVO, and gray dots indicate genes that were not differentially expressed from our analysis. D) MA plot of *ovo^ΔBP^/ovo^ovo-GAL4^*; *UASp-3xFHA-OVO-B* versus *ovo^ΔBP^/ovo^ovo-GAL4^*; *UASp-GFP* RNA-seq differential expression results. Cyan dots indicate genes that significantly increased in expression and were found to be moderately expressed in 0-2 hour old embryos, yellow dots indicate genes that significantly decreased in gene expression and were found to be moderately expressed in 0-2 hour old embryos, purple dots indicate genes that were not differentially expressed and were found to be moderately expressed in 0-2 hour old embryos, and gray dots indicate genes that were not differentially expressed and were not found to be moderately expressed in 0-2 hour old embryos. E) Gene level read coverage heatmaps of OVO ChIP minus input, GSC and 32c ATAC-seq, GSC H3K27ac, H3K4me3, H3K27me3, H3K9me3, 8c NC H3K9me3, 32c NC H3K27ac, and H3K27me3 ChIP-seq, and *ovo^ΔBP^/ovo^ovo-GAL4^*; *UASp-3xFHA-OVO-B* minus *ovo^ΔBP^/ovo^ovo-GAL4^*; *UASp-GFP* RNA-seq for genes bound by OVO.

Occupancy is a requirement for activity, but occupancy does not equal activity. Understanding the transcriptional consequences of OVO occupancy genome-wide would allow us to investigate mechanisms. However, as we mentioned earlier, the fact that *ovo* is absolutely required for female germline viability greatly complicates this analysis. Measuring gene expression in dead or dying germ cells was unlikely to be informative. Luckily, one *ovo* mRNA isoform with an extended exon 2 is also required for egg chamber development beyond stage 5 (Salles et al. 2002; Benner et al. 2023). Utilizing this finding allowed us to determine which genes in the female germline are transcriptionally responsive to OVO during oogenesis. To do this, we used our *ovo^ovo-GAL4^* allele. *ovo^ovo-GAL4^* is a hypomorphic allele that truncates the translation of *ovo-B* transcripts that include the exon 2 extension. About 90% of *ovo* mRNAs are of this type (Benner et al. 2023). Females trans-heterozygous for *ovo^ovo-GAL4^* and the *ovo-B* null allele *ovo^ΔBP^* show germline survival, with surviving germ cells arresting around stage 5 of oogenesis (Figure 4A)(Benner et al. 2023). Since this allele inherently expresses GAL4 in place of full length OVO due to the T2A sequences, we can drive expression of rescuing and control sequences downstream with *UASp* to generate excellent control samples. Therefore, we compared the gene expression occurring in sets of ovaries that had the *ovo* hypomorphic phenotype with a sham rescue construct (*ovo^ovo-GAL4^*/*ovo^ΔBP^*; *UASp-GFP*)(Figure 4A) versus those that drive the expression of the rescue construct expressing OVO-B (*ovo^ovo-GAL4^*/*ovo^ΔBP^*; *UASp-3xFHA-OVO-B*)(Figure 4B).

Since *ovo^ovo-GAL4^*/*ovo^ΔBP^*; *UASp-3xFHA-OVO-B* females have full rescue of the arrested germ cell phenotype seen in *ovo^ovo-GAL4^*/*ova^ΔBP^*; *UASp-GFP*females, we needed to take further measures to ensure our analysis of gene expression was stage comparable between the two sets of ovaries. We therefore chose to dissect one day post-eclosion *ovo^ovo-GAL4^*/*ova^ΔBP^*; *UASp-3xFHA-OVO-B* female ovaries to enrich for early stages of oogenesis, and collected only ovarioles containing the germarium through previtellogenic egg chambers. *ovo^ovo-GAL4^*/*ovo^ΔBP^*; *UASp-GFP* ovaries were collected at the same age post-eclosion and we specifically collected ovaries that contained a visible ovariole structure (and therefore contained germ cells) to minimize comparing germ cells to somatic ovary structures, but rather germ cells to germ cells. We then performed RNA-seq in quadruplicate and measured the changes in gene expression between the two genotypes. We used a significance level of p-adj < 0.05 and a log2 fold change cutoff of >|0.5| to call differential expression. We utilized these log2 fold change cutoffs for two reasons. Our control ovary genotype (*ovo^ovo-GAL4^*/*ovo^ΔBP^*; *UASp-GFP*) has hypomorphic OVO activity, hence germ cells are able to survive but are arrested. With the addition of ectopic rescue OVO in *ovo^ovo-GAL4^*/*ovo^ΔBP^*; *UASp-3xFHA-OVO-B* ovaries, we predicted that genes that were directly regulated by OVO would transcriptionally respond, however, we were unsure as to what degree the response would be in comparison to hypomorphic OVO. So we therefore wanted to use a less conservative log2 fold change cutoff in order to not miss any expression changes between hypomorphic OVO and rescue OVO. We reasoned that if the changes were not significant between genotypes then minor changes in gene expression would not matter. Our second reason for using these cutoffs is we had an internal control between the two genotypes. We knew through immunostaining that Vas protein was present in the germline of both genotypes (Figure 4A, 4B) and therefore was likely expressed at similar levels in the germline of both genotypes. Both genotypes also expressed *GAL4* under the control of *ovo* in the germline. We examined the expression levels of *vas* and *GAL4* and found that *vas* had a log2 fold change of 0.15 (p-adj = 0.03) and *GAL4* had a log2 fold change of 0.33 (p-adj = 0.18) (Figure 4C). These data suggest a slight underrepresentation of germline expression in the ovo mutant ovaries. Therefore, by using the greater than 0.5 and less than −0.5 log2 fold change cutoffs, and a less than 0.05 p-adj value cutoff, we would be conservative to not call genes differentially expressed due to differences in the relative abundance of germ cells and somatic cells.

We were able to reliably detect the expression of 10,804 genes in these early ovarioles (Table S5). The differential expression analysis indicated that 1,994 genes primarily expressed in the germline (see next paragraph) significantly increased in expression with ectopic rescue OVO (Figure 4C, cyan/purple dots) and 2,924 genes primarily expressed in the soma (see next paragraph) significantly decreased in expression with ectopic rescue OVO expression (Figure 4C, yellow/blue dots). 5,886 genes were not considered to be differentially expressed in our analysis (Figure 4C, gray dots). OVO is expressed in the germline, not the soma, and previous work has shown that OVO-B is a transcriptional activator (Andrews et al. 2000), so we hypothesized that many of the genes increasing in gene expression in the presence of rescuing OVO were direct downstream targets. As a test, we intersected OVO-dependent gene expression with OVO occupancy data. Among genes that showed OVO-dependent expression, we found a significant enrichment for genes with OVO ChIP peaks (Figure 4C, cyan dots, p < 0.01, odds ratio = 2.21) while conversely, there was a significant depletion of genes that showed decreased expression in OVO rescue ovaries and OVO ChIP peaks (Figure 4C, blue dots, p < 0.01, odds ratio = 0.85). This strongly suggests that genes that are bound by OVO, transcriptionally respond in a positive manner. This finding is fully consistent with our meta-analysis comparing OVO ChIP-seq and histone ChIP-seq/ATAC-seq data (Figure 4E). OVO binding was highly associated with transcriptionally active histone marks such as H3K27ac and H3K4me3, open chromatin, and increased expression.

There are genes that showed decreased expression in the OVO rescued ovaries, but we believe this is technical rather than biological. OVO is expressed only in the germline, but ovarioles contain germ cells and somatic cells. The presence of empty ovarioles, containing leftover strings of somatic cells, are evident even in *ovo^ovo-GAL4^*/*ovo^ΔBP^*; *UASp-GFP* ovaries that contain germ cells. Conversely, *ovo^ovo-GAL4^*/*ovo^ΔBP^*; *UASp-3xFHA-OVO-B* ovaries are fully rescued, and therefore possess more germ cell containing ovarioles than *ovo^ovo-GAL4^*/*ovo^ΔBP^*; *UASp-GFP* ovaries (Benner et al. 2023). Despite our best efforts to dissect individual ovarioles with a full complement of germ cells and egg chambers, we wondered if there might be fewer germ cells and chambers in the *ovo^ovo-GAL4^*/*ovo^ΔBP^*; *UASp-GFP* ovaries. To address this directly, we tested the idea that genes with significantly increased expression with the presence of ectopic rescue OVO were enriched for germ cell specific expression, and thus downstream of OVO. We also tested the idea that genes that significantly decreased in expression were due to somatic cell lineages, which should be enriched in the *ovo* hypomorph due to reduced numbers of germ cells in the ovarioles (Benner et al. 2023). To confirm that genes increasing in expression in ectopic rescue OVO were germline derived, we cross-referenced the significantly expressed genes in our RNA-seq datasets with the modENCODE developmental RNaseq datasets (Graveley 2010). We extracted the gene names of all genes that were considered to be ‘moderately expressed’ in 0-2 hour old embryos, which are produced during oogenesis and are deposited into the early embryo. We found that 71% of genes (1,409/1,994) that had a significant increase in expression in the presence of ectopic rescue OVO were found to be expressed in 0-2 hour old embryos (Figure 4D, green dots), while only 21% of genes (625/2,924) that had a significant decrease in expression were found in the same embryo dataset (Figure 4D, yellow dots). 3,448 genes from the 0-2 hour old embryo dataset were not differentially expressed in our RNA-seq dataset (Figure 4D, purple dots). A Fisher’s exact test confirmed that there was a significant enrichment for genes that significantly increased in expression and were present in 0-2 hour old embryos (p < 0.01, odds ratio = 2.8). In comparison, there was a significant depletion for genes that significantly decreased in expression and were present in 0-2 hour old embryos (p < 0.01, odds ratio = 0.17). This result indicated that genes that significantly increased in expression were more likely to be expressed in the germline and that the presence of ectopic rescue OVO significantly increased the expression of genes that were maternally deposited in the early embryo. While the set of genes that significantly decrease in expression are not enriched in the embryo and are more likely specific to somatic cell gene expression. These genes are unlikely to be direct OVO targets due to the absence of OVO in those cells, although we certainly cannot rule out the possibility of a non-autonomous effect of OVO on somatic gene expression. In terms of the germline proper, OVO appears to be a positively acting transcription factor.

### OVO Positively Regulates Essential Oogenesis Genes

**Figure 5:**
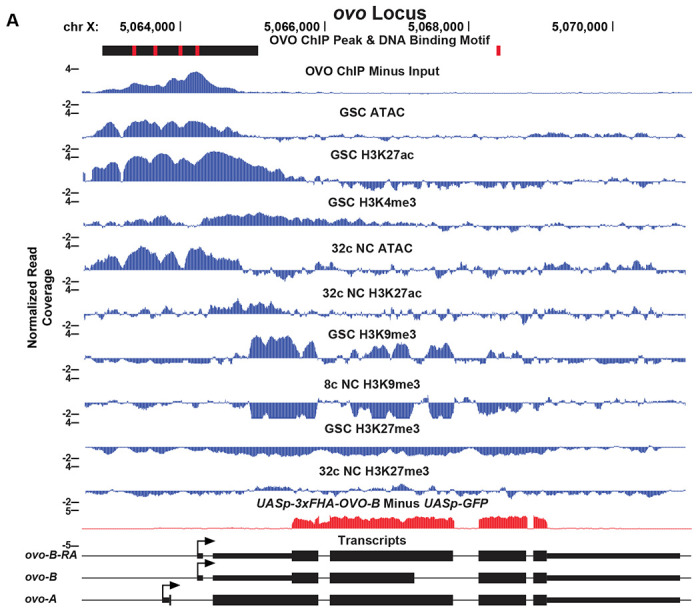
OVO ChIP-seq, ATAC/Histone ChIP-seq, RNA-seq, and DNA Binding Motifs at the *ovo* Locus. *ovo* gene level read coverage tracks for OVO ChIP minus input, GSC and 32c ATAC-seq, GSC H3K27ac, H3K4me3, H3K27me3, H3K9me3, 8c NC H3K9me3, 32c NC H3K27ac, and H3K27me3 ChIP-seq, and *ovo*^*ΔBP*^/*ovo*^*ovo-GAL4*^; *UASp-3xFHA-OVO-B* minus *ovo*^*ΔBP*^*/ovo^ovo-GAL4^*; *UASp-GFP* RNA-seq. Red rectangles and black rectangles represent significant OVO DNA binding motifs and OVO ChIP peaks, respectively. Gene models are represented at bottom. Small rectangles represent untranslated regions, large rectangles represent translated regions. Arrows indicate transcriptional start sites.

We wanted to examine a subset of the OVO target genes in detail, and began with the known OVO targets, *ovo* itself and *otu* (Bielinska et al. 2005; Lü et al. 1998; Lü and Oliver 2001). Since the relationship between OVO binding to these two genes has been well-characterized, we validated the OVO ChIP, histone ChIP/ATAC-seq, and RNA-seq datasets by examining these two genes first. Since OVO positively regulates the expression of both these genes, then we would expect OVO to be physically bound at OVO motifs required for high transcription, the presence of transcriptionally active histone marks and open chromatin, as well as a positive transcriptional response in the presence of rescuing OVO-B. This is exactly what we observed. A significant OVO ChIP peak was found overlapping the TSS of *ovo-B*, with four significant OVO DNA binding motifs present (Figure 5A). ATAC-seq, H3K27ac, and H3K4me3 peaks overlapped the *ovo* promoter. Transcriptionally, *ovo* RNA-seq reads are derived from the *UASp-3xFHA-OVO-B* cDNA rescue, so we could not assess whether *ovo* responded transcriptionally to ectopic rescue OVO. However, when looking at the *otu* locus (Figure S2), we found OVO occupancy over the TSS of both annotated *otu* promoters, with significant OVO DNA binding motifs overlapping and in close proximity to the TSSs. The *otu* locus contained similar ATAC-seq and activating histone mark peaks overlapping the TSS found at the *ovo* locus. It was also evident that *otu* had a positive transcriptional response to the presence of OVO rescue (log2 fold change = 2.41; p-adj < 0.001). These results confirm that OVO binds and positively regulates both itself and *otu in-vivo*, as previous work has indicated.

**Figure 6:**
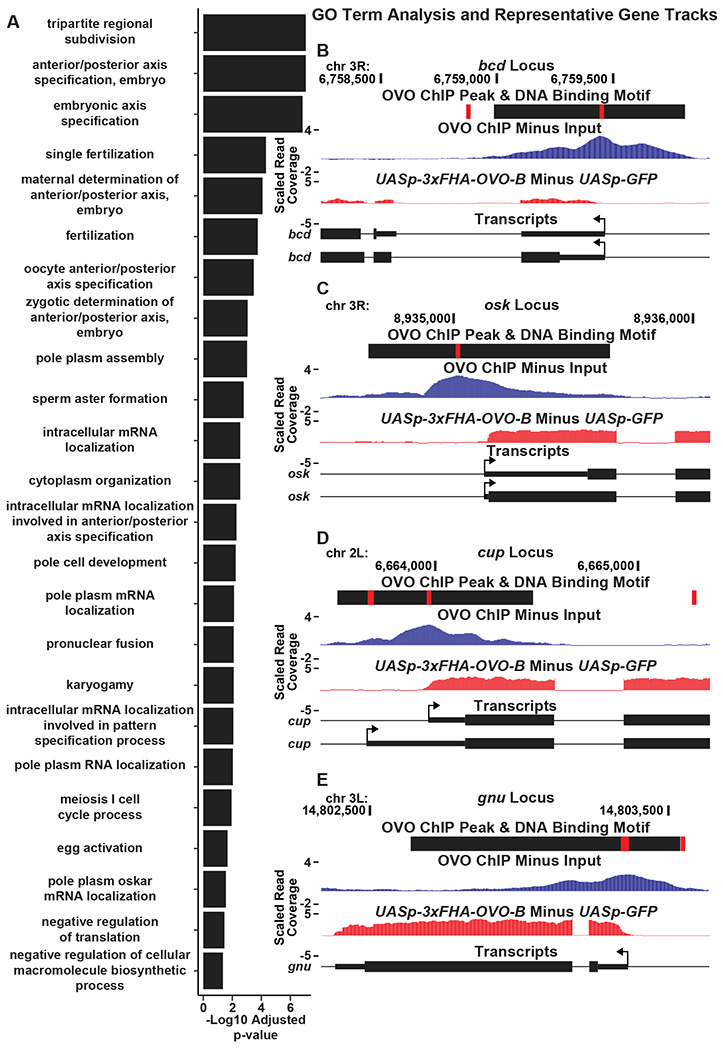
OVO Binds and Significantly Increases the Expression of a Number of Genes Involved in Essential Maternal Processes. A) Significantly enriched GO biological process terms for genes bound by OVO and significantly increase in expression in the presence of ectopic rescue OVO. GO terms are restricted to terms containing less than 125 associated genes. B-E) Example GO term gene level read coverage tracks for OVO ChIP minus input and *ovo*^*ΔBP*^*/ovo^ovo-GAL4^*; *UASp-3xFHA-OVO-B* minus *ovo*^*ΔBP*^*/ovo*^*ovo-GAL4*^; *UASp-GFP*. Red rectangles and black rectangles represent significant OVO DNA binding motifs and OVO ChIP peaks, respectively. Gene models are represented at bottom. Small rectangles represent untranslated regions, large rectangles represent translated regions. Arrows indicate transcriptional start sites.

Since our overlapping OVO ChIP-seq and RNA-seq data suggests that hundreds of genes that are bound by OVO increase in expression in the presence of ectopic rescue OVO, we wanted to know more about the functions of those genes. For the big picture, we performed Gene Ontology enrichment analysis with gProfiler software (Raudvere et al. 2019). To be especially stringent, we focused on the genes that contained an OVO ChIP peak overlapping the transcriptional start site and significantly increased in expression in the presence of rescue OVO. 525 genes met these criteria. Biological process GO term enrichment analysis on these 525 genes showed a significant enrichment for 109 GO terms (Table S6). The significant GO terms were almost exclusively related to female reproduction and maternal control of early embryonic development, including embryonic axis specification, mRNA localization, egg activation, and negative regulation of translation (Figure 6A). These associated GO terms are well understood in the context of oogenesis and broadly suggest that OVO expression in adult gonads is essential for constructing an egg and depositing maternal RNAs to support zygotic embryonic development.

GO term enrichment analysis of genes that are bound by OVO and increase in expression in the presence of ectopic rescue OVO, suggested that OVO is likely a main transcriptional regulator of oogenesis. These genes are the subject of decades of work on *Drosophila* oogenesis, but essentially all the work on them has focused on what they do, not on how they are transcriptionally regulated. For example, *bicoid* (Figure 4B), and *bicoid* mRNA binding proteins *exuperantia* (*exu*) and *swallow* (*swa*), are essential for anterior specification of the embryo (Lasko 2012). All of these genes were occupied by OVO *in vivo*, significantly upregulated by OVO-B, and had OVO motifs in close proximity to the TSS. Genes involved in posterior patterning (*oskar* and *nanos*) (Figure 4C), as well as pole cell specification genes (*polar granule component, germ cell-less*, and *aubergine*)(Lasko 2012; L. Benner, Deshpande, and Lerit 2018), also showed similar RNA-seq, ChIP-seq, and OVO DNA binding motif profiles as *ovo* and *otu*. Genes that are involved in translational silencing and regulation of maternally provided mRNAs, such as *cup* (Figure 4D), *maternal expression at 31B (me31B), oo18 RNA-binding protein (orb)*, and *bruno 1* (*bru1*) as well as essential genes involved in meiosis completion and egg activation after fertilization (*giant nuclei* (*gnu*), *pan gu* (*png*), *plutonium* (*plu*), *wispy* (*wisp*), *C(3)G*, and *matrimony* (*mtrm*)) (Figure 4E)(Lasko 2012; Avilés-Pagán and Orr-Weaver 2018) all show this stereotypic pattern of promoter proximal OVO occupancy and DNA binding motifs, and OVO-dependent transcription. These data indicate that the *ovo* locus is a central transcription factor activating the expression of essential maternal and early embryonic development pathways in the female germline.

We also found that the genes *fs(1)N, fs(1)M3*, and *closca*, were all bound by OVO and responded transcriptionally to the presence of ectopic rescue OVO. These genes are significant because they constitute a set of genes that are expressed in the germline and are eventually incorporated into the vitelline membrane providing the structural integrity and impermeability of the egg (Mineo, Furriols, and Casanova 2017; Ventura et al. 2010). Loss-of-function of these three genes results in flaccid eggs that are permeable and fail to develop. A testament to the likelihood of OVO regulating these genes is the finding that females transheterozygous for the dominant antimorphic *ovo^D3^* allele lay eggs that are flaccid and contain a permeable vitelline membrane, assayed through their ability to uptake the dye neutral red (Oliver, Pauli, and Mahowald 1990). Presumably in these females, the expression of the repressive form of OVO leads to a reduction in the expression of these three essential vitelline membrane genes which phenocopies loss-of-function alleles of each, respectively. Altogether, this largely indicates that not only is OVO involved in regulating the expression of numerous essential maternal pathways for embryonic development, it is also essential for regulating genes that are required for egg integrity and maturation.

## Discussion

Since its original isolation as a dominant female sterile locus in *Drosophila* (Busson et al. 1983; Komitopoulou et al. 1983), *ovo* has long been known as an essential gene in oogenesis. Female germ cells require *ovo* for survival and differentiation, while it has no described roles or functions in the male germline (Oliver, Perrimon, and Mahowald 1987; Oliver, Pauli, and Mahowald 1990). OVO has also been found to be eternally present in the female germline, attesting to its likely continual requirement for female germ cell viability and identity (Benner et al. 2023). Our work here significantly expands our knowledge on OVO function in the female germline, showing that OVO binds and positively regulates a large array of genes required to build an egg and pattern the resulting embryo after fertilization. OVO accomplishes this by directly binding to the promoters of its targets, as well as more distant sites that could represent enhancers. Altogether, we suggest that OVO is a master transcriptional regulator coordinating a number of essential maternal pathways involved in oocyte and early embryonic development. Hints of these functions can be found in the hypomorphic and antimorphic *ovo* alleles which show egg chamber arrest, ventralized eggs, and permeable vitelline membranes. It is clear that OVO is required to activate multiple pathways involved in oocyte and early embryonic development.

The GO term enrichment analysis on genes that were bound by OVO and transcriptionally responded to OVO surprisingly indicated a large degree of overlap in oocyte and early embryonic developmental pathways. Also, OVO seemed to reinforce these pathways at multiple key genes within each pathway. For example, OVO bound to the promoters and increased the expression of *bcd*, and the *bcd* mRNA binding proteins *exu* and *swa*, all involved in ensuring correct anterior specification of the embryo (Lasko 2012). Genes that are essential for egg activation were coordinately regulated by OVO as well. OVO downstream target genes *gnu, png, plu*, and *wisp* all belong in the same interconnected pathway ensuring egg activation (Avilés-Pagán and Orr-Weaver 2018). A similar story was found for genes such as *cup, Me31b, bru1*, and *orb*, indicating that OVO controls a battery of genes involved in the positive regulation of RNA binding proteins that negatively regulate translation (Lasko 2012). OVO also bound and positively regulated a number of posterior and germ plasm specification genes such as *osk, nos, aub, gcl*, and *pgc* (Lasko 2012; Mahowald 2001). Given this plethora of famous maternal effect loci, it might be tempting to suggest that OVO is sufficient for egg production, but there are important exceptions. For example, other important germ plasm factors such as *vasa, staufen*, and *tudor*were not bound by or transcriptionally responsive to OVO. This observation suggest that other transcription factors are be responsible for regulating these genes.

OVO binds in close proximity to the TSS of genes it positively regulates, however, it is still unclear precisely how it regulates gene expression. The possibilities include integration into the RNA Polymerase complex itself, a short distance sigma factor like function, a core promoter conditioning function (pioneering), and/or garden variety transcription factor. Although core promoters active specifically in the ovary are enriched for OVO DNA binding motifs, we did not find a strict spatial orientation for these motifs in relation to the TSS, such as is found with other DNA elements such as INR, DPE, and MTE (Ohler et al. 2002; FitzGerald et al. 2006; Lim et al. 2004). It is therefore unlikely that OVO is a core component of the RNA Polymerase complex in the female germline. This suggests that it is unlikely to be analogous to male specific TATA-associated factors that have been shown to activate gene expression in the male germline (M. Hiller et al. 2004; M. A. Hiller et al. 2001; Lu et al. 2020; V. C. Li et al. 2009). It is therefore possible, and previously well supported, that OVO is a strict activator of transcription (Lü et al. 1998; Lü and Oliver 2001; Bielinska et al. 2005). Where OVO binding in close proximity, in any orientation relative to the TSS, activates transcription. One aspect of OVO DNA binding that showed differences with stage-specific histone ChIP and ATAC-seq, was OVO’s strong association with open chromatin. The role of repressors of transcription such as the polycomb complex, *egg, wde*, and *Su(var)205* in restricting gene expression through promoting heterochromatin formation in differentiating egg chambers is well established (Smolko, Shapiro-Kulnane, and Salz 2018; DeLuca et al. 2020). OVO might ensure that the chromatin status of maternally expressed genes remains open. Evidence from our work points to OVO fulfilling that role.

In GSCs and early egg chambers, OVO ChIP peaks largely overlap open chromatin and transcriptionally active histone marks. However, in stage 5 egg chambers, there was an even higher degree of association with open chromatin (all OVO ChIP peaks overlapped ATAC-seq peaks), while the significant association with H3K27ac marks was greatly reduced. This difference is likely significant. As GSCs differentiate, they accumulate repressive chromatin marks while the number of ATAC-seq and H3K27ac peaks are reduced. This increase in association with open chromatin and OVO binding, even as the amount of open chromatin is reduced throughout egg chamber differentiation, might indicate that OVO binding helps to maintain chromatin accessibility, even when the locus in question is no longer actively transcribed. The loss of histone marks of active transcription at OVO-bound open chromatin in later differentiating egg chambers might mean that OVO does not influence the transcriptional potential of target genes as strongly as it influences the chromatin status in this second phase. Therefore, OVO might be more similar in function to pioneer factors/chromatin remodelers than it is to a transcription factor that is only involved in activating transcription.

In light of OVOs stronger association with open chromatin over histone marks identified with active transcription, evidence for OVO as a potential pioneering factor comes through its developmental protein expression and the eventual expression of downstream target genes. OVO is maternally deposited into the embryo where it eventually is sequestered to embryonic pole cells (Hayashi et al. 2017; Benner et al. 2023). Maternal OVO maintains a nuclear localization within the germline throughout embryonic development until it eventually overlaps with its own zygotic gene expression as well as its downstream target *otu* at stage 15 (Benner et al. 2023; Casper and Van Doren 2009). Pole cells are transcriptionally quiescent (A. Nakamura and Seydoux 2008; Deshpande et al. 1999; Van Doren, Williamson, and Lehmann 1998), with the first signs of zygotic transcription occurring at stage 8 (Van Doren, Williamson, and Lehmann 1998). So why doesn’t zygotic *ovo* and *otu* transcription begin until much later in development even though maternal OVO is present and germ cells are transcriptionally competent much earlier? One explanation for this is that OVO bound genes are also dependent on other transcription factors in the developing germline, despite being remarkably compact. The OVO-dependent *ovo* core promoter is very compact; showing expression with only 10s of bp of sequence in reporters. While it is possible that OVO is co-dependent on the presence of other transcription factors that are expressed later, or that OVO does not have the ability to activate transcription by itself and replaced by a more traditional transcription factor. This role is more similar to pioneer transcription factors such as *zelda* (*zld*).

**Figure 7:**
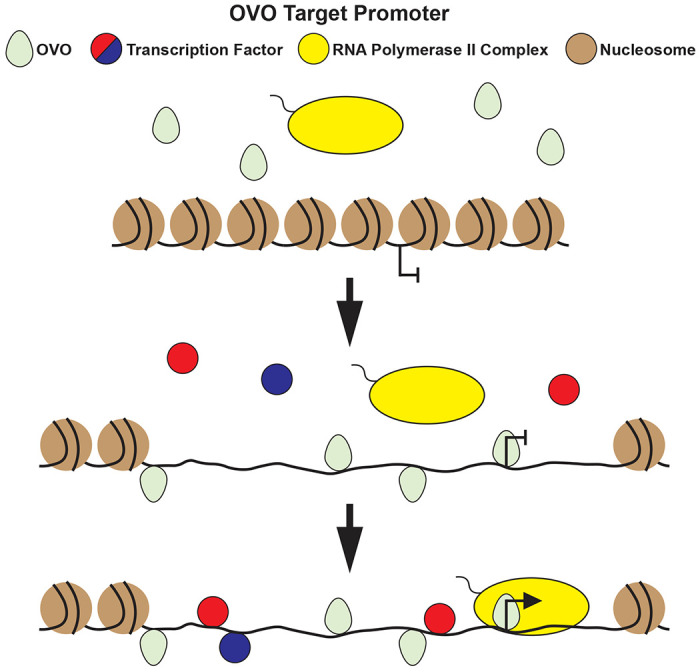
Proposed Model for OVO Regulation in the Female Germline Throughout Development. Embryonic pole cells, which are transcriptionally quiescent, contain maternally deposited nuclear OVO. As development ensues and embryonic germ cells become transcriptionally competent, maternal OVO binds to its target promoters but does not positively influence gene expression at this stage. Later in embryonic development, in the presence of other activating transcription factors, maternal OVO works in concert to positively influence gene expression, activating a number of essential germ cell-specific genes.

It has been well established that *zld* influences the maternal to zygotic transition by binding its target genes before they become transcriptionally active (Harrison et al. 2011). ZLD binding increases chromatin accessibility which allows for other activating transcription factors to bind and positively influence gene expression (Xu et al. 2014). Loss of maternal *zld* does affect a target promoter’s ability to be activated, indicating that the difference between a pioneer factor and a transcriptional activator is slight, but reducing chromatin accessibility can negate the effects of the presence of a transcriptional activator and its ability to influence a target promoter. Although pioneer factors are expressed in the female germline and are maternally deposited into the embryo to regulate the maternal to zygotic transition, surprisingly, a functional role for pioneer factors has yet to be described in the developing embryonic germline. If OVO is involved in influencing chromatin status, then maternally depleting it should have a negative effect on embryonic germ cell chromatin status and gene expression. At this time, it is technically challenging to deplete maternal OVO and determine the embryonic germ cell phenotype, as attempts to deplete maternal OVO leads to egg chamber arrest/death in adult females. However, based on OVO’s relationship to open chromatin, its eternal expression in the female germline, and delayed target gene expression, we propose a model where maternal OVO binds target promoters and influences chromatin status in the transcriptionally quiescent embryonic germline. OVO binding a target promoter without the presence of other transcription factors, however, does not in itself activate gene expression. As germ cells develop, the eventual expression of activating transcription factors allow for OVO bound genes to become expressed (Figure 7). Expression of OVO bound genes are required in the adult germline to allow for germ cell differentiation and egg development. Ultimately resulting in the maternal deposition of mRNAs and proteins required for early embryonic development, including *ovo* mRNA and OVO protein, maintaining the cyclical nature of the germline.

## Supplementary Material

Supplement 1 Table S1: Flybase ART Table.

Supplement 2 Table S2: OVO ChIP-seq Statistics by Chromosome.

Supplement 3 Table S3: Genomic Locations of Significant Overlapping OVO ChIP Peaks

Supplement 4 Table S4: Significant OVO DNA Binding Motifs in MEME Format

Supplement 5 Table S5: Differential Expression Analysis Results for All Genes

Supplement 6 Table S6: Significantly Enriched GO Biological Process Terms

7

## Data Availability

Drosophila strains used for this study are available upon request. All sequence information and datasets used in this study are in Table S1.
