# Supplementary material for: OVO Positively Regulates Essential Maternal Pathways by Binding Near the Transcriptional Start Sites in the Drosophila Female Germline
Source: bioRxiv. 2024 Apr 30:2023.11.01.565184. Originally published 2023 Nov 4. Preprint. [Version 3] doi: 10.1101/2023.11.01.565184 (PMC10705541; doi:10.1101/2023.11.01.565184)
Supplement: Supplement 7 [file NIHPP2023.11.01.565184v3-supplement-7.pdf]

bioRxiv preprint doi: https://doi.org/10.1101/2023.11.01.565184; this version posted April 30, 2024. The copyright holder for this preprint (which was not certified by peer review) is the author/funder. This article is a US Government work. It is not subject to copyright under 17 USC 105 and is also made available for use under a CC0 license.

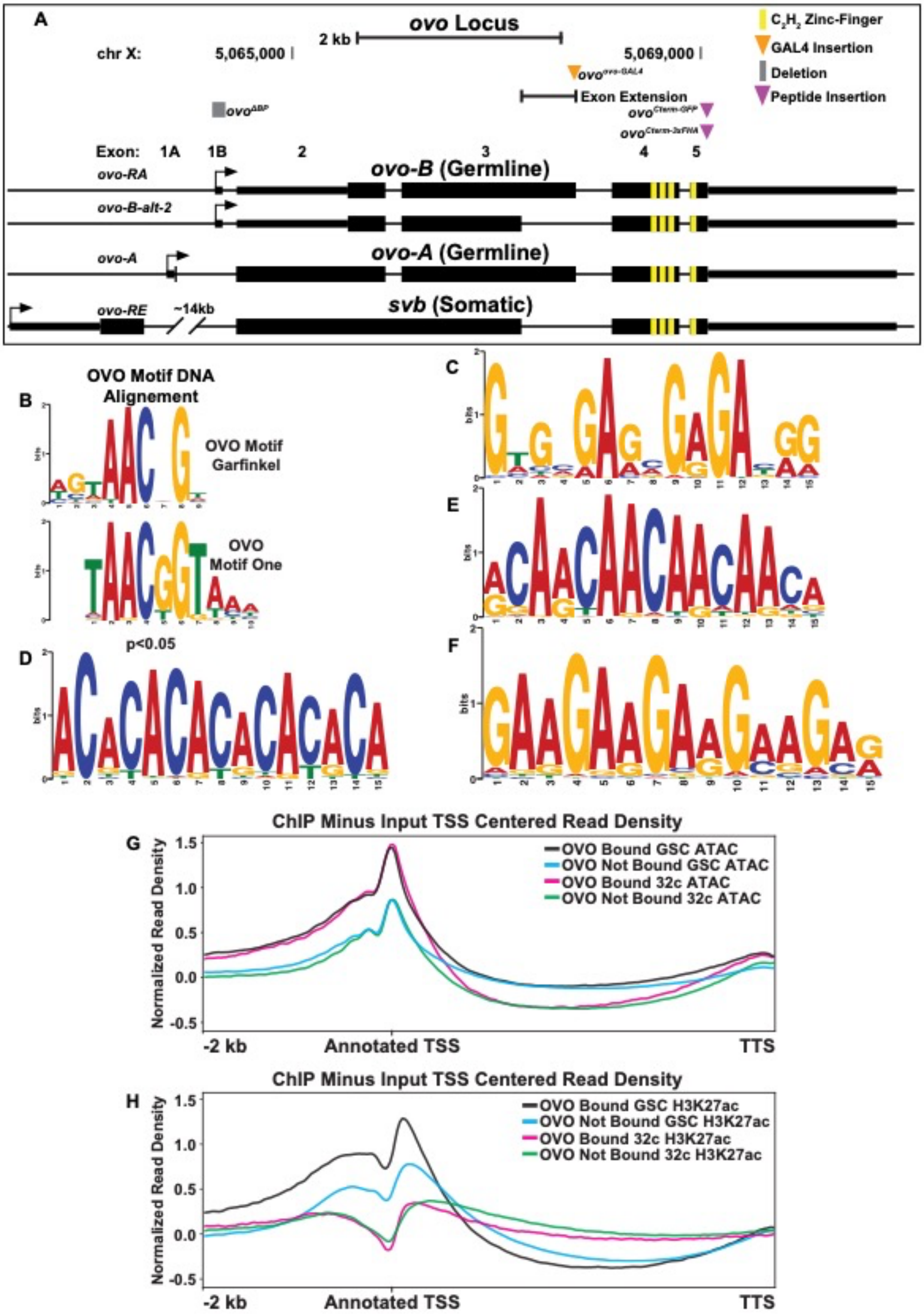

1113

bioRxiv preprint doi: https://doi.org/10.1101/2023.11.01.565184; this version posted April 30, 2024. The copyright holder for this preprint (which was not certified by peer review) is the author/funder. This article is a US Government work. It is not subject to copyright under 17 USC 105 and is also made available for use under a CC0 license.

Figure S1: Significant OVO DNA Binding Motif. A) Cartoon of the transcripts expressed from the *ovo* locus based on Benner et al. 2023. Gray box represents endogenously generated deletions, orange triangle represents the location of the *T2A-GAL4-3xSTOP* insertion, purple triangles represent the location of small peptide insertions and yellow boxes represent the location of the shared C_2H_2 zinc-fingers. Small rectangles represent untranslated regions, large rectangles represent translated regions and arrows indicate TSSs. B) Significant alignment of the *in vivo* OVO DNA binding ‘motif one’ and *in vitro* OVO DNA binding motif (Lee and Garfinkel 2000). C-F) Significantly enriched DNA motifs within OVO ChIP peaks. C is a signficiant match for the DNA binding motif of GAF and CLAMP (Omelina et al. 2011; Soruco et al. 2013). G, H. GSC and 32c ATAC-seq and H3K27ac ChIP-seq minus input control read coverage density for genes bound by OVO over the TSS, gene body, closest TSS in intergenic space, closest TSS for all, or not bound. Plots are centered on the TSS.

bioRxiv preprint doi: https://doi.org/10.1101/2023.11.01.565184; this version posted April 30, 2024. The copyright holder for this preprint (which was not certified by peer review) is the author/funder. This article is a US Government work. It is not subject to copyright under 17 USC 105 and is also made available for use under a CC0 license.

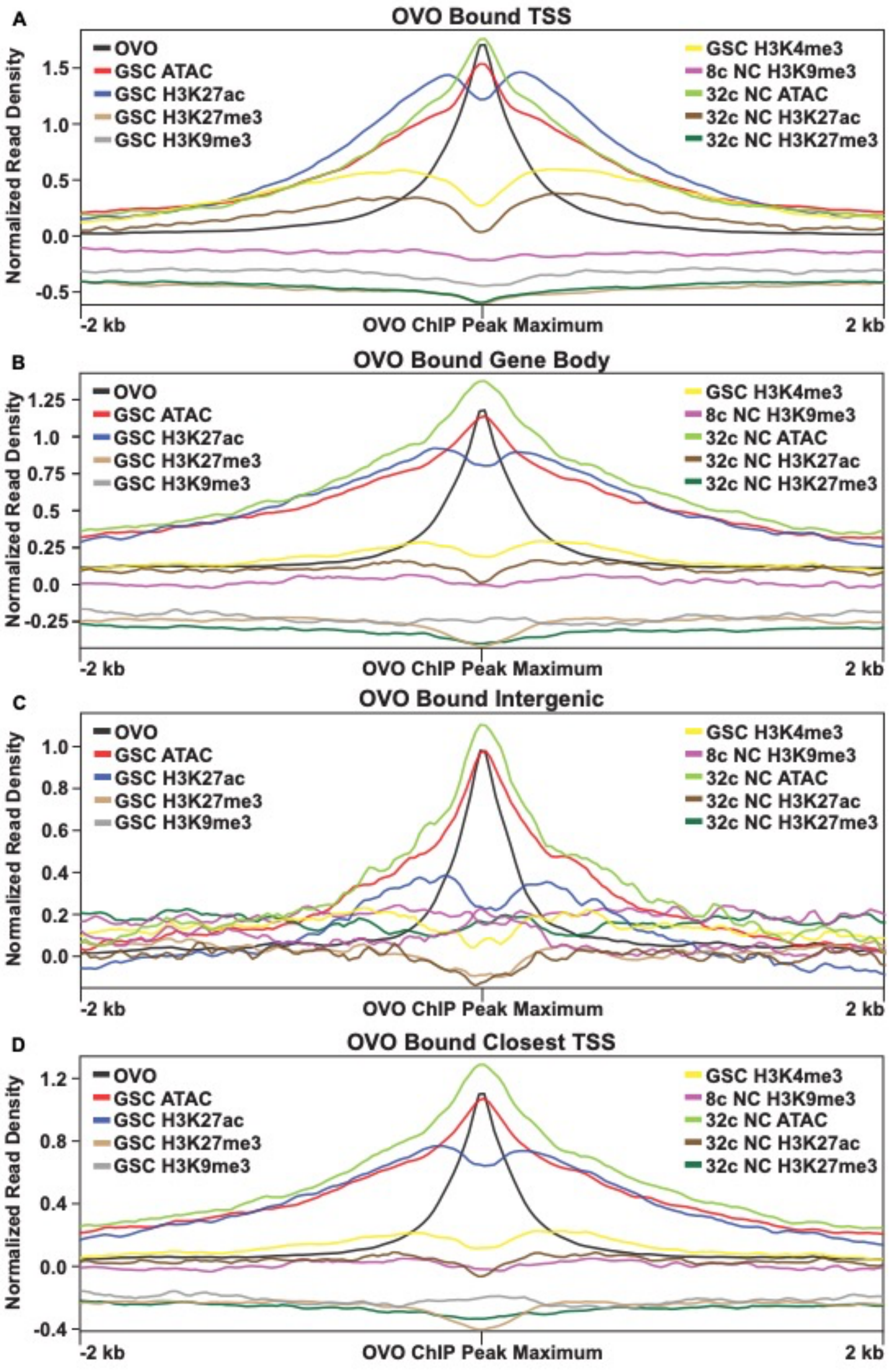

1125

bioRxiv preprint doi: https://doi.org/10.1101/2023.11.01.565184; this version posted April 30, 2024. The copyright holder for this preprint (which was not certified by peer review) is the author/funder. This article is a US Government work. It is not subject to copyright under 17 USC 105 and is also made available for use under a CC0 license.

Figure S2: OVO DNA Binding is Associated with Open Chromatin and Transcriptionally Active Histone Marks Across Variations of Gene Binding Patterns. A-D) OVO ChIP minus input control, GSC and 32c ATAC-seq, GSC H3K27ac, H3K4me3, H3K27me3, H3K9me3, 8c NC H3K9me3, 32c NC H3K27ac, and H3K27me3 ChIP-seq read coverage density centered on OVO peak maximums located within a significant OVO ChIP peak for genes bound by OVO over the TSS, gene body, closest TSS in intergenic space, or closest TSS for all.

bioRxiv preprint doi: https://doi.org/10.1101/2023.11.01.565184; this version posted April 30, 2024. The copyright holder for this preprint (which was not certified by peer review) is the author/funder. This article is a US Government work. It is not subject to copyright under 17 USC 105 and is also made available for use under a CC0 license.

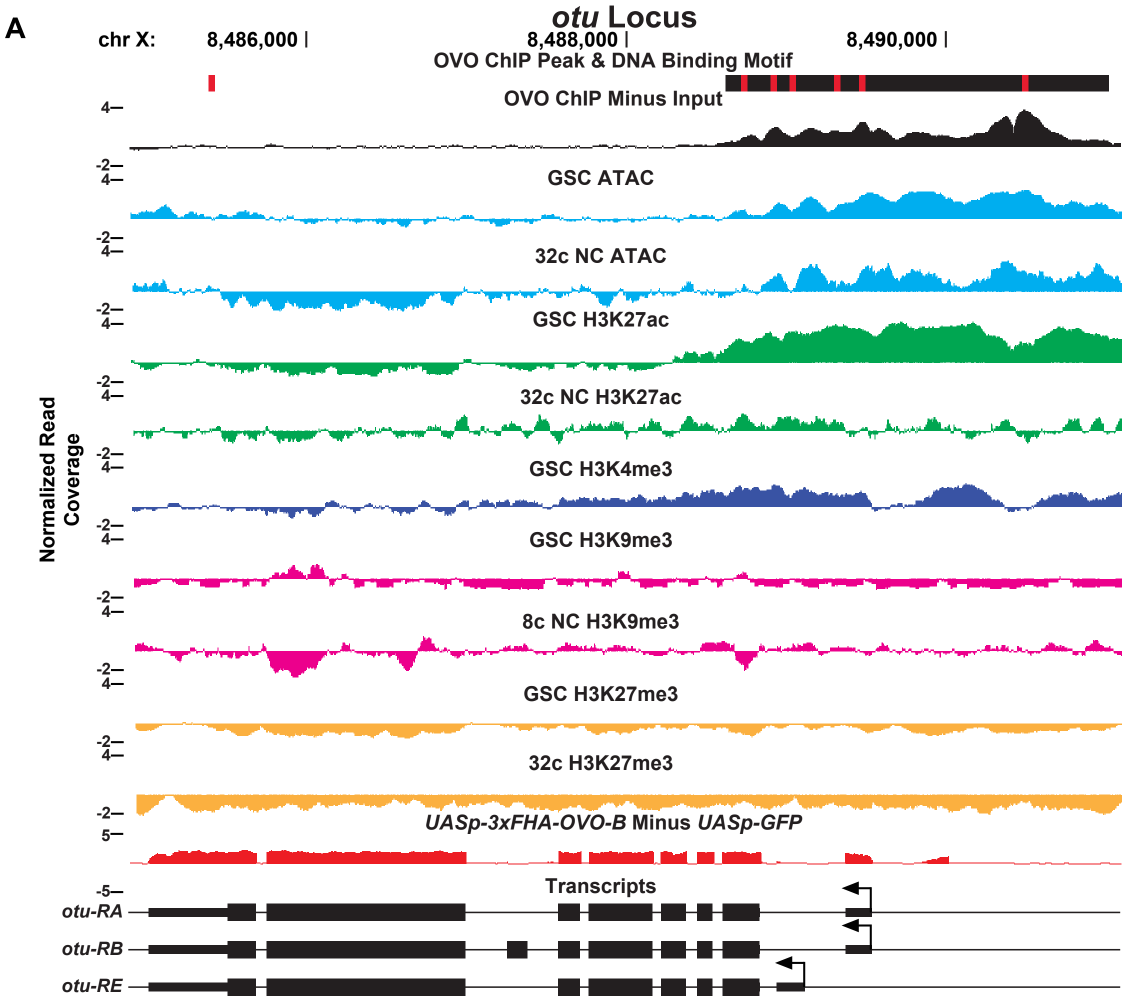

1132

bioRxiv preprint doi: https://doi.org/10.1101/2023.11.01.565184; this version posted April 30, 2024. The copyright holder for this preprint (which was not certified by peer review) is the author/funder. This article is a US Government work. It is not subject to copyright under 17 USC 105 and is also made available for use under a CC0 license.

Figure S3: OVO ChIP-seq, ATAC/Histone ChIP-seq, RNA-seq, and DNA Binding Motifs at the *otu* Locus. A) *otu* gene level read coverage tracks for OVO ChIP minus input (black), GSC and 32c ATAC-seq (light blue), GSC and 32C H3K27ac (green), H3K4me3 (dark blue), GSC and 32c H3K27me3 (orange), and GSC and 8c H3K9me3 (pink) ChIP-seq, and $ovo^{\Delta BP}/ovo^{ovo\text{-}GAL4}$; *UASp-3xFHA-OVO-B* minus $ovo^{\Delta BP}/ovo^{ovo\text{-}GAL4}$; *UASp-GFP* RNA-seq (red). Red rectangles and black rectangles represent significant OVO DNA binding motifs and OVO ChIP peaks, respectively. Gene models are represented at bottom. Small rectangles represent untranslated regions, large rectangles represent translated regions. Arrows indicate transcriptional start sites.
