# Supplementary material for: OVO Positively Regulates Essential Maternal Pathways by Binding Near the Transcriptional Start Sites in the Drosophila Female Germline
Source: bioRxiv. 2023 Dec 8:2023.11.01.565184. Originally published 2023 Nov 4. Preprint. [Version 2] doi: 10.1101/2023.11.01.565184 (PMC10705541; doi:10.1101/2023.11.01.565184)
Supplement: Supplementary file 1 [file NIHPP2023.11.01.565184v2-supplement-7.pdf]

bioRxiv preprint doi: https://doi.org/10.1101/2023.11.01.565184; this version posted December 8, 2023. The copyright holder for this preprint (which was not certified by peer review) is the author/funder. This article is a US Government work. It is not subject to copyright under 17 USC 105 and is also made available for use under a CC0 license.

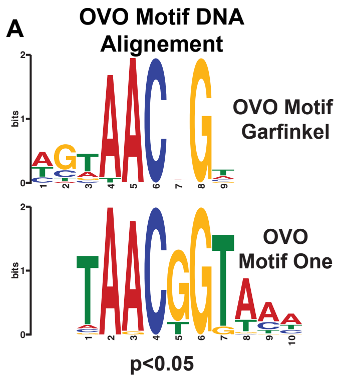

Figure S1: Significant OVO DNA Binding Motif Matches and *da-GAL4* X UAS Transgene Viability. A) Significant alignment of the *in vivo* OVO DNA binding 'motif one' and *in vitro* OVO DNA binding motif (OVO Garfinkel).

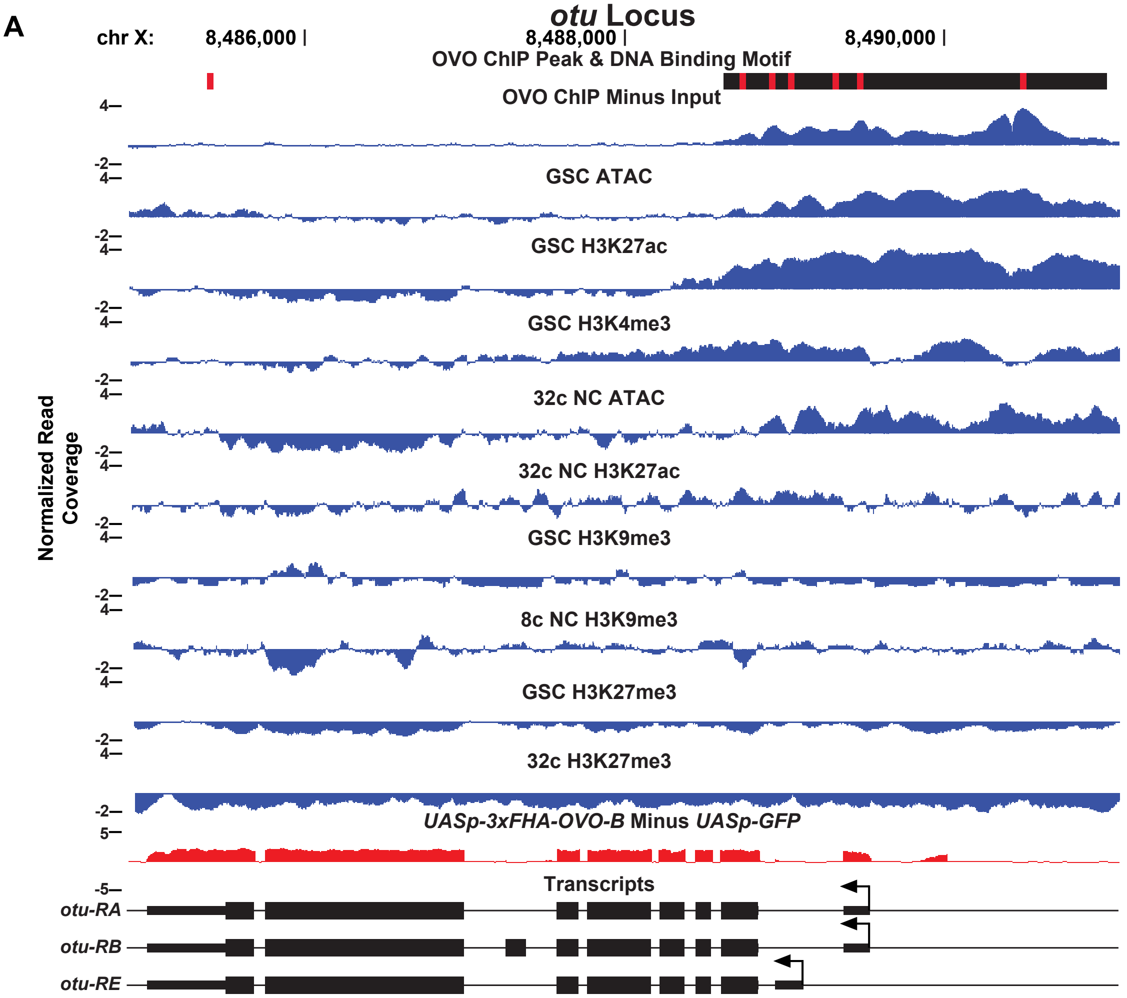

Figure S2: OVO ChIP-seq, ATAC/Histone ChIP-seq, RNA-seq, and DNA Binding Motifs at the *otu* Locus. A) *otu* gene level read coverage tracks for OVO ChIP minus input, GSC and 32c ATAC-seq, GSC H3K27ac, H3K4me3, H3K27me3, H3K9me3, 8c NC H3K9me3, 32c NC H3K27ac, and H3K27me3 ChIP-seq, and $ovo^{\Delta BP}/ovo^{ovo\text{-}GAL4}$; *UASp-3xFHA-OVO-B* minus $ovo^{\Delta BP}/ovo^{ovo\text{-}GAL4}$; *UASp-GFP* RNA-seq. Red rectangles and black rectangles represent significant OVO DNA binding motifs and OVO ChIP peaks, respectively. Gene models are represented at bottom. Small rectangles represent untranslated regions, large rectangles represent translated regions. Arrows indicate transcriptional start sites.
